# A Phase 3 Clinical Study to Evaluate the Safety, Tolerability, and Immunogenicity of V116 in Pneumococcal Vaccine–Experienced Adults 50 Years of Age or Older (STRIDE-6)

**DOI:** 10.1093/cid/ciae383

**Published:** 2024-07-31

**Authors:** Paul Scott, Miwa Haranaka, Jung Hyun Choi, Helen Stacey, Marc Dionne, David Greenberg, Carlos G Grijalva, Walter A Orenstein, Doreen Fernsler, Nancy Gallagher, Tiantian Zeng, Jianing Li, Heather L Platt, for the STRIDE-6 Study Group, Timothy J Chapman, Karyn Davis, Marc Dionne, Peter Dzongowski, Ginette Girard, Guy Tellier, Richard Tytus, Sylvain Jaffuel, Jean-Francois Nicolas, Eytan Ben Ami, Daniele Bendayan, Yoseph Caraco, Michal Chowers, Mahmud Darawsha, Avivit Peer, Francesco Bruno Blasi, Antonella Castagna, Claudio Costantino, Domenico Martinelli, Miwa Haranaka, Makoto Yono, Jung Hyun Choi, Won Suk Choi, Dong-Gun Lee, Jacob Lee, Hyejin Shi, Joon Young Song, Gustavo De luiz Martinez, Jose Maria Echave-Sustaeta Maria-Tome, Cristina Masuet Aumatell, Silvia Narejos Perez, Anna Vilella i Morato, Kuo-Chin Huang, Yi-Ching Yang, David J Butuk, Jose Francisco Cardona, Nizar Daboul, Thomas Fiel, Neil J Fraser, George Hartley Freeman, Steven A Geller, Charles Harold Harper, William Henry Johnston, Thomas C Lenzmeier, Enrique Pelayo, Laura Porterfield, Kathryn R Rigonan, Jeffrey Bruce Rosen, Helen L Stacey

**Affiliations:** Merck & Co., Inc., Rahway, New Jersey, USA; SOUSEIKAI PS Clinic, Fukuoka, Japan; Catholic University of Korea, Seoul, South Korea; Diablo Clinical Research, Walnut Creek, California, USA; Université Laval, Quebec City, Quebec, Canada; Soroka University Medical Center, Beer-Sheva, Israel; Vanderbilt University Medical Center, Nashville, Tennessee, USA; Emory University, Atlanta, Georgia, USA; Merck & Co., Inc., Rahway, New Jersey, USA; Merck & Co., Inc., Rahway, New Jersey, USA; Merck & Co., Inc., Rahway, New Jersey, USA; Merck & Co., Inc., Rahway, New Jersey, USA; Merck & Co., Inc., Rahway, New Jersey, USA

**Keywords:** vaccine, pneumococcal, adult, safety, V116

## Abstract

**Background:**

Pneumococcal diseases cause considerable morbidity and mortality in adults. V116 is an investigational 21-valent pneumococcal conjugate vaccine (PCV) specifically designed to protect adults from pneumococcal serotypes responsible for the majority of residual pneumococcal diseases. This phase 3 study evaluated safety, tolerability, and immunogenicity of V116 in pneumococcal vaccine–experienced adults aged ≥50 years.

**Methods:**

A total of 717 adults were enrolled to receive a single dose of pneumococcal vaccine as follows: cohort 1 (n = 350) previously received 23-valent pneumococcal polysaccharide vaccine (PPSV23) and were randomized 2:1 to receive V116 or PCV15, respectively; cohort 2 (n = 261) previously received PCV13 and were randomized 2:1 to receive V116 or PPSV23, respectively; cohort 3 (n = 106) previously received PPSV23 + PCV13, PCV13 + PPSV23, PCV15 + PPSV23, or PCV15 and all received open-label V116. Immunogenicity was evaluated 30 days postvaccination using opsonophagocytic activity (OPA) geometric mean titers (GMTs) and immunoglobulin G (IgG) geometric mean concentrations (GMCs) for all V116 serotypes. Safety was evaluated as the proportion of participants with adverse events (AEs).

**Results:**

V116 was immunogenic across all 3 cohorts as assessed by serotype-specific OPA GMTs and IgG GMCs postvaccination for all 21 serotypes. V116 elicited comparable immune responses to serotypes shared with PCV15 (cohort 1) or PPSV23 (cohort 2), and higher immune responses to serotypes unique to V116. The proportions of participants with solicited AEs were generally comparable across cohorts.

**Conclusions:**

V116 is well tolerated with a safety profile comparable to currently licensed pneumococcal vaccines and generates IgG and functional immune responses to all V116 serotypes, regardless of prior pneumococcal vaccine received.

**Clinical Trials Registration:**

NCT05420961; EudraCT 2021-006679-41.

## Teaser

V116, an investigational 21-valent pneumococcal conjugate vaccine (PCV), was immunogenic for all vaccine serotypes in adults previously vaccinated with a pneumococcal vaccine, with a safety profile generally comparable to 15-valent PCV and 23-valent pneumococcal polysaccharide vaccine. Study results support the use of V116 in adults.

Disease due to infection with *Streptococcus pneumoniae* is a serious public health issue, with millions affected globally each year. Infants, older adults, and adults with medical comorbidities including the immunocompromised are most vulnerable to pneumococcal disease (PD). In adults, *Streptococcus pneumoniae* is the leading bacterial cause of community-acquired pneumonia (CAP) [1–5], which is associated with considerable morbidity and mortality.

Pneumococcal vaccines have proven to be highly effective at reducing the overall burden of PD when used as part of national immunization programs. As a result of childhood vaccination, the burden of disease in children has decreased substantially, with a concurrent decrease, though more modest, in adults. Direct vaccination of adults has provided protection as well, and due to both direct and indirect effects of these vaccination programs, the serotypes responsible for the majority of PD in adults have changed over time. Due to serotype replacement, serotypes not covered by currently available vaccines are responsible for much of the residual PD burden. Therefore, there is a need for continued pneumococcal vaccine development to reduce the burden of PD. Prior research has shown that pneumococcal serotypes commonly detected in adults with PD are not the same as those detected among children with PD [6]. In recent studies of CAP as well as hospitalized individuals with invasive PD (IPD) such as meningitis and bacteremia, serotypes 3, 19A, 22F, and 9N contained in currently licensed vaccines and serotypes 15A, 23B, 24F, 35B, and others not currently covered by a licensed vaccine contributed to the residual burden of disease in adults [7–12].

Current guidelines for adult pneumococcal vaccination from the Advisory Committee on Immunization Practices (United States [US]) recommend that adults 19–64 years of age with at-risk conditions and adults ≥65 years of age should receive 15-valent pneumococcal conjugate vaccine (PCV15) followed by 23-valent pneumococcal polysaccharide vaccine (PPSV23), or 20-valent pneumococcal conjugate vaccine (PCV20) alone [13]. Further, adults who received only PPSV23 may receive a PCV (either PCV20 or PCV15) ≥1 year after their PPSV23 dose, while those who previously received 13-valent pneumococcal conjugate vaccine (PCV13) should complete the vaccination series with a dose of PPSV23.

V116 (Merck, Sharp & Dohme LLC, a subsidiary of Merck & Co., Inc., Rahway, New Jersey, USA [MSD]) is a PCV designed specifically for adults and is approved for the prevention of IPD and pneumonia in adults ≥18 years of age [14]. V116 contains 21 serotypes (3, 6A, 7F, 8, 9N, 10A, 11A, 12F, 15A, 15C, 16F, 17F, 19A, 20A, 22F, 23A, 23B, 24F, 31, 33F, 35B) that are associated with the majority of residual IPD among adults ≥65 years of age in countries with established pediatric national immunization programs. In the US, serotypes covered by V116 caused approximately 85% of IPD in adults aged ≥65 years prior to the coronavirus disease 2019 (COVID-19) pandemic. From the same data set, approximately 30% of the IPD coverage from V116 serotypes comes from 8 serotypes unique to V116 that are not included in a currently licensed pneumococcal vaccine [15]. Some changes in pneumococcal serotype epidemiology have occurred since the COVID-19 pandemic, but overall vaccine serotype distribution has remained consistent [16–20]. For the US, data through 2022 shows that serotypes covered by V116 still caused 81% of IPD in adults aged 18–49 years with risk-based indications and 85% of IPD in all adults ≥65 years of age [20]. Early phase trials of V116 evaluated its safety and tolerability, as well as its immunogenicity against all vaccine serotypes in pneumococcal vaccine–naive adult populations [21, 22]. This phase 3 study evaluated safety, tolerability, and immunogenicity of V116 in adults ≥50 years of age who had previously received PCV15, PPSV23, or a combination of approved pneumococcal vaccines.

## METHODS

### Study Design

This was a phase 3, double-blind (cohorts 1 and 2), open-label (cohort 3), multicenter, active comparator, parallel assignment study that evaluated the safety, tolerability, and immunogenicity of V116 in pneumococcal vaccine–experienced adults ≥50 years of age (protocol number V116–006). The study was conducted at 51 centers in 9 countries (Supplementary Table 1) from July 2022 to June 2023 (ClinicalTrials.gov NCT05420961 and EudraCT 2021-006679-41).

The study was designed to enroll 700 participants into 3 parallel cohorts based on the participant's pneumococcal vaccination history. Cohorts 1 and 2 were double-blind and active comparator controlled, and cohort 3 was open label. Cohort 1 was targeted to enroll 300 participants vaccinated with PPSV23 ≥1 year prior to study enrollment, then randomized 2:1 to receive V116 or PCV15 on study day 1, respectively. Cohort 2 was targeted to enroll 300 participants vaccinated with PCV13 ≥1 year prior to study enrollment, then randomized 2:1 to receive V116 or PPSV23 on study day 1, respectively. Cohort 3 was targeted to enroll 100 participants vaccinated with PCV13 + PPSV23, PCV15 + PPSV23, PCV15, PCV20, or PPSV23 + PCV13 ≥1 year prior to study enrollment, then allocated to receive V116 on study day 1. Treatment allocation/randomization occurred centrally using an interactive response technology system and was stratified (for cohorts 1 and 2 only) according to (1) participant age at enrollment and (2) time since last pneumococcal vaccination.

Study vaccines were prepared and administered by unblinded, qualified study site personnel who were not involved in subsequent participant assessments or study procedures. An external data monitoring committee conducted periodic safety review for the study. A scientific advisory committee comprised of external and MSD scientists contributed to the development of the protocols and statistical analysis plan. The study was conducted in accordance with the principles of Good Clinical Practice and approved by the appropriate institutional review boards/ethics committees.

### Participants

Participants were enrolled at clinical academic or research sites. Eligible participants included those ≥50 years of age who were not pregnant or breastfeeding and were pneumococcal vaccine experienced, defined as prior receipt of an eligible pneumococcal vaccination regimen ≥1 year before enrollment. History of pneumococcal vaccination was verified by the investigator using source documents including vaccination records and clinical notes. Medical and medication histories were collected by participant self-report. Written informed consent was obtained from each participant prior to any study procedure.

Key exclusion criteria were as follows: (1) history of IPD or other culture-positive pneumococcal disease within 3 years of start of the study; (2) known hypersensitivity to any component of PCV15, PCV20, or PPSV23; (3) known or suspected impairment of immune function (including but not limited to: history of congenital or acquired immunodeficiency, documented HIV infection, functional or anatomic asplenia, or history of autoimmune disease) or currently receiving immunosuppressive therapy; (4) febrile illness or receipt of antibiotic therapy within 72 hours of receipt of any study vaccine; or (5) received PPSV23 followed by either PCV15 or PCV20 prior to study enrollment.

### Vaccines and Administration

V116 (MSD) is a 21-valent PCV. Each 0.5-mL dose contains 4 µg of each pneumococcal capsular polysaccharide (PnPs): 3, 6A, 7F, 8, 9N, 10A, 11A, 12F, 15A, deOAc15B, 16F, 17F, 19A, 20A, 22F, 23A, 23B, 24F, 31, 33F, and 35B, all individually conjugated to CRM197. The deOAc15B PnPs is hereafter referred to as 15C as it is structurally similar to the 15C PnPs and antibodies to 15C were evaluated. PCV15 and PPSV23 are both licensed pneumococcal vaccines (MSD). Study vaccines were supplied as prefilled syringes and stored at 2°C–8°C. A 0.5-mL intramuscular dose of V116, PCV15, or PPSV23 was administered, and participants were observed for at least 30 minutes postvaccination.

### Safety Assessments

Participants used an electronic vaccination report card to record daily maximum body temperatures and solicited injection-site (erythema, pain, swelling) and systemic (fatigue, headache, myalgia) adverse events (AEs) through day 5 after vaccination. Other AEs were reported for 30 days postvaccination, and serious AEs (SAEs) and deaths were reported from day 1 through the duration of participation in the study. All recorded complaints were reviewed by study investigators to determine if they met protocol-defined AE criteria and to assess seriousness, intensity, and causality to the study vaccine.

### Immunogenicity Assessments

Blood was collected on day 1 prior to study vaccination and at day 30 for measurement of serotype-specific anti-PnPs antibodies. Functional antibodies were measured using a serotype-specific microcolony multiplex opsonophagocytic killing assay. Serotype-specific anti-PnPs immunoglobulin G (IgG) antibodies were evaluated with a multiplex electrochemiluminescence assay using custom plates from Meso Scale Discovery (Rockville, Maryland). Both assays were developed at MSD [23, 24].

### Determination of Study Sample Size

The primary goal of the study was to evaluate the safety, tolerability, and immunogenicity of V116 in individuals with various pneumococcal vaccination histories. The study objectives are descriptive, and no formal hypothesis testing was performed. The sample size of 200 in each of cohorts 1 and 2 and 100 in cohort 3 receiving V116 was selected to achieve a reasonably sized safety database in this population. For instance, in cohorts 1 and 2 there is an 80% chance of observing at least 1 SAE among 200 participants if the underlying incidence of a SAE is 0.8%. If no SAEs are observed among 200 participants, the study will provide 97.5% confidence that the underlying percentage of participants with a SAE is <1.83%.

### Safety Endpoints and Statistical Methods

The primary safety endpoint was to evaluate the safety and tolerability of V116 as assessed by the proportion of participants with AEs, including solicited injection-site AEs, solicited systemic AEs, and vaccine-related SAEs. For each AE category, estimated within-group 95% confidence intervals (CIs) were calculated based on the exact method by Clopper and Pearson [25]. Safety analyses were conducted on the “all participants as treated” population, which consisted of all randomized participants who received at least 1 study vaccination.

### Immunogenicity Endpoints and Statistical Methods

The primary immunogenicity objective was to evaluate the serotype-specific opsonophagocytic activity (OPA) geometric mean titers (GMTs) at 30 days postvaccination for all V116 serotypes. Secondary objectives were to evaluate the serotype-specific IgG geometric mean concentrations (GMCs), as well as the geometric mean fold rise (GMFR) and proportion of participants with a ≥4-fold rise in serotype-specific antibodies from baseline (day 1) to 30 days postvaccination for all V116 serotypes. For the continuous endpoints, point estimates were calculated by exponentiating the estimates of the mean of the natural log values and the within-group CIs were derived by exponentiating the CIs of the mean of the natural log values based on the t-distribution. For the dichotomous endpoints, within-group CIs were calculated based on the exact method proposed by Clopper and Pearson. Immunogenicity analyses were conducted on the “per protocol” population, which consisted of all randomized participants without protocol deviations that may substantially affect results of the immunogenicity endpoints.

### Analysis Software

All analyses were performed using SAS software, version 9.4, of the SAS System for Unix (©2012 SAS Institute, Inc).

## RESULTS

**Figure 1.**
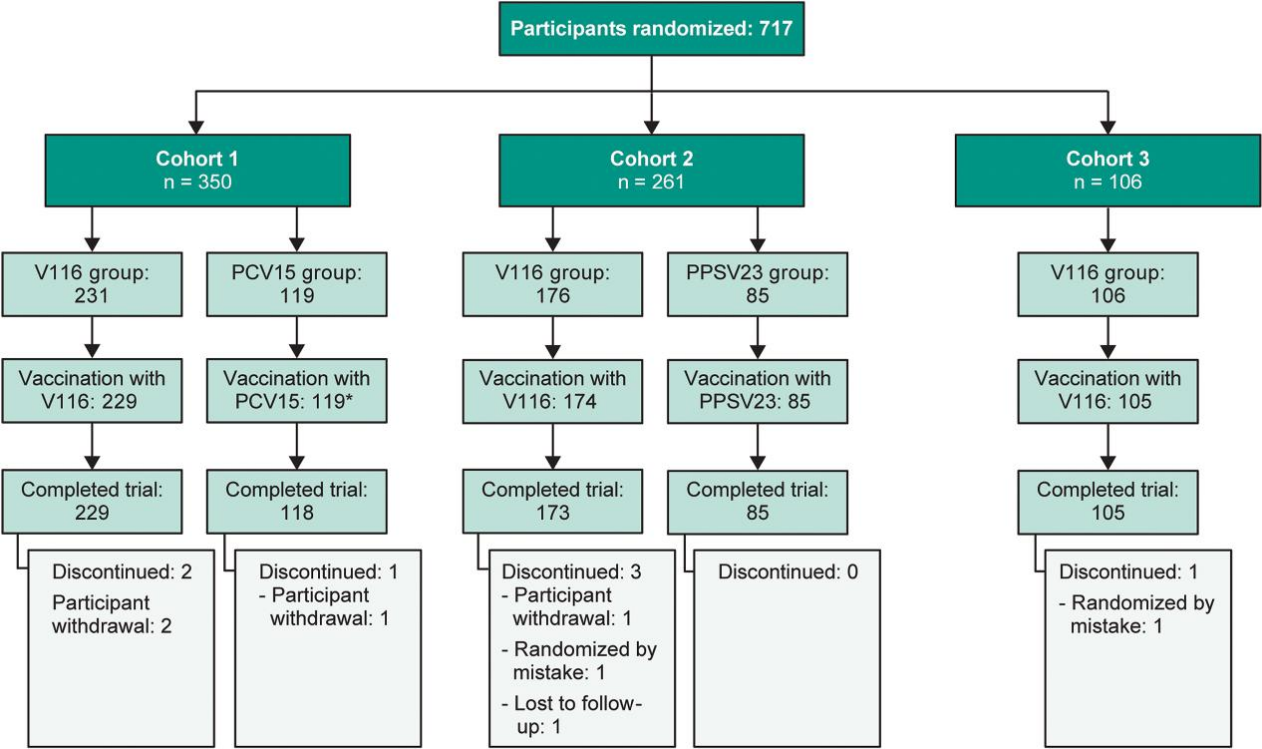
Participant disposition. Participants were assigned to 3 cohorts based on pneumococcal vaccine experience: prior vaccination with 23-valent pneumococcal polysaccharide vaccine (PPSV23; cohort 1), prior vaccination with 13-valent pneumococcal conjugate vaccine (PCV13; cohort 2), or prior vaccination with PCV13 + PPSV23, 15-valent pneumococcal conjugate vaccine (PCV15) + PPSV23, PCV15, 20-valent pneumococcal conjugate vaccine (PCV20), or PPSV23 + PCV13 (cohort 3). *One hundred seventeen participants received PCV15, and 2 were cross-vaccinated with 21-valent pneumococcal conjugate vaccine (V116) and PPSV23 (1 each).

A total of 717 participants ≥50 years of age were assigned to the 3 cohorts, and 710 (99.0%) completed the study (Figure 1). Participants were well balanced across cohorts for sex, race, and ethnicity, with the majority of participants in all cohorts being female and White, non-Hispanic (Table 1). Asian, Black or African American, and Hispanic or Latino participants were represented in all cohorts. The majority of participants (70.6%) were ≥65 years of age (Table 1). Approximately 47% of all vaccinated participants had 1 or more prespecified medical history conditions (alcoholism, chronic heart disease, chronic kidney disease, chronic liver disease, chronic lung disease, diabetes, and/or smoking) associated with an increased risk of PD. These participants were similarly distributed across cohorts (Supplementary Table 2). The majority of participants across cohorts had received a pneumococcal vaccination within the last 4 years. Cohort 1 had a larger proportion of participants who received their last pneumococcal vaccination 5 or more years prior to study enrollment (Table 1).

**Table 1.** Baseline Demographics and Clinical Characteristics

Characteristic	Cohort 1 (n = 348)		Cohort 2 (n = 259)		Cohort 3 (n = 105)
	V116 (n = 229)	PCV15 (n = 119)	V116 (n = 174)	PPSV23 (n = 85)	V116 (n = 105)
Sex, No. (%)					
Male	112 (48.9)	59 (49.6)	74 (42.5)	36 (42.4)	50 (47.6)
Female	117 (51.1)	60 (50.4)	100 (57.5)	49 (57.6)	55 (52.4)
Age, y, No. (%)					
50–64	48 (21.0)	25 (21.0)	80 (46.0)	39 (45.9)	17 (16.2)
≥65	181 (79.0)	94 (79.0)	94 (54.0)	46 (54.1)	88 (83.8)
Mean	68.7	69.0	65.5	65.4	71.0
Race, No. (%)					
Asian	96 (41.9)	47 (39.5)	55 (31.6)	25 (29.4)	13 (12.4)
Black or African American	6 (2.6)	3 (2.5)	3 (1.7)	1 (1.2)	6 (5.7)
Multiple	2 (0.9)	0 (0.0)	0 (0.0)	0 (0.0)	1 (1.0)
White	125 (54.6)	69 (58.0)	116 (66.7)	59 (69.4)	85 (81.0)
Ethnicity, No. (%)					
Hispanic or Latino	21 (9.2)	17 (14.3)	34 (19.5)	16 (18.8)	14 (13.3)
Not Hispanic or Latino	206 (90.0)	102 (85.7)	140 (80.5)	69 (81.2)	91 (86.7)
Not reported	1 (0.4)	0 (0.0)	0 (0.0)	0 (0.0)	0 (0.0)
Unknown	1 (0.4)	0 (0.0)	0 (0.0)	0 (0.0)	0 (0.0)
Time since last pneumococcal vaccination, No. (%)					
1–4 y	108 (47.2)	54 (45.4)	135 (77.6)	66 (77.6)	78 (74.3)
5–9 y	85 (37.1)	45 (37.8)	33 (19.0)	18 (21.2)	27 (25.7)
≥10 y	36 (15.7)	20 (16.8)	6 (3.4)	1 (1.2)	0 (0.0)

Number of participants enrolled by country can be found in Supplementary Table 1.

Abbreviations: PCV15, 15-valent pneumococcal conjugate vaccine; PPSV23, 23-valent pneumococcal polysaccharide vaccine; V116, 21-valent pneumococcal conjugate vaccine.

### Immunogenicity

**Figure 2.**
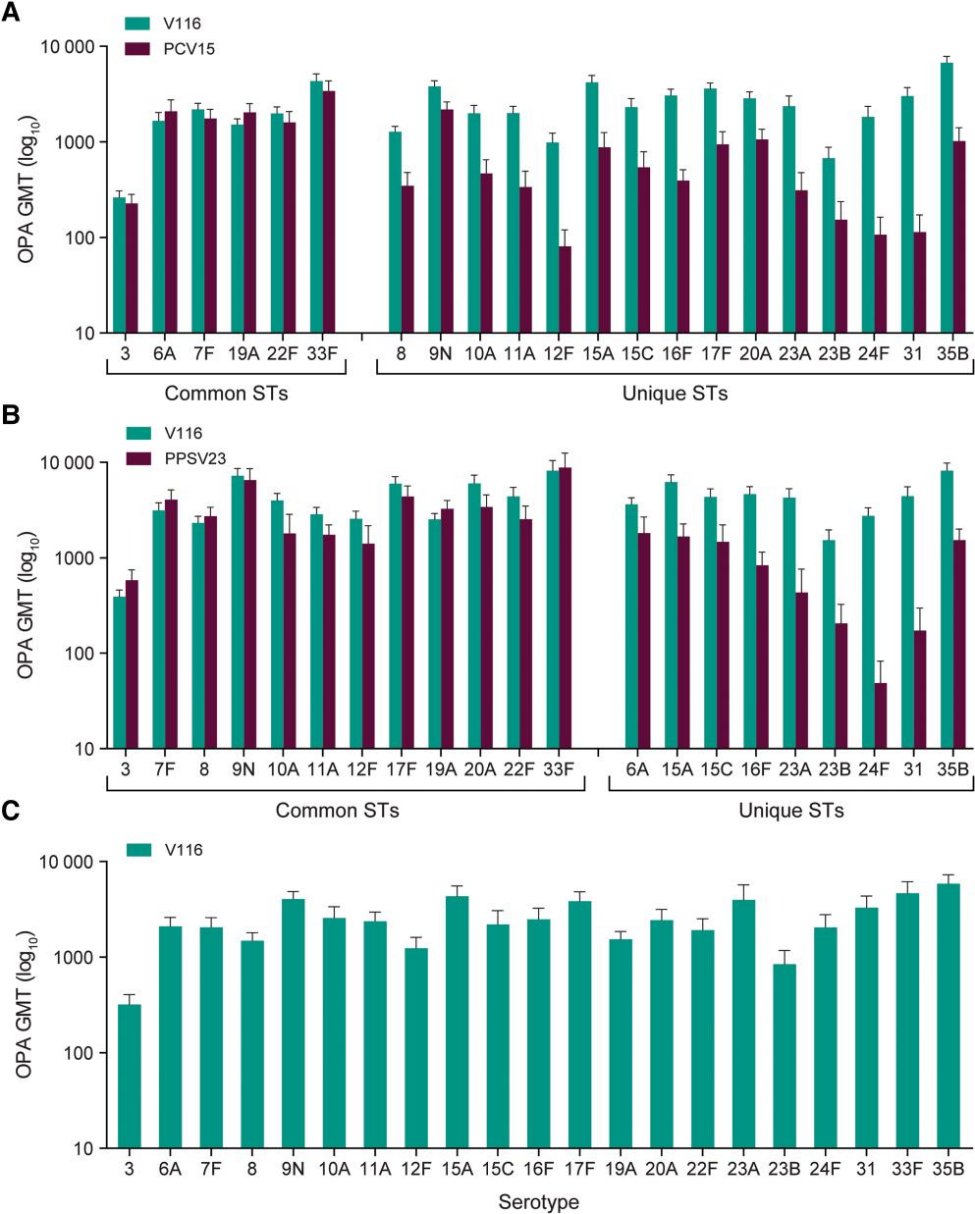
Serotype-specific opsonophagocytic activity (OPA) geometric mean titers (GMTs) at 30 days following vaccination. OPA GMTs and 95% confidence intervals are shown for each serotype by cohort (*A*, cohort 1; *B*, cohort 2; *C*, cohort 3), with serotypes shared in both vaccines or unique to V116 shown on the x-axis. Abbreviations: GMT, geometric mean titer; OPA, opsonophagocytic activity; PCV15, 15-valent pneumococcal conjugate vaccine; PPSV23, 23-valent pneumococcal polysaccharide vaccine; ST, serotype; V116, 21-valent pneumococcal conjugate vaccine.

**Figure 3.**
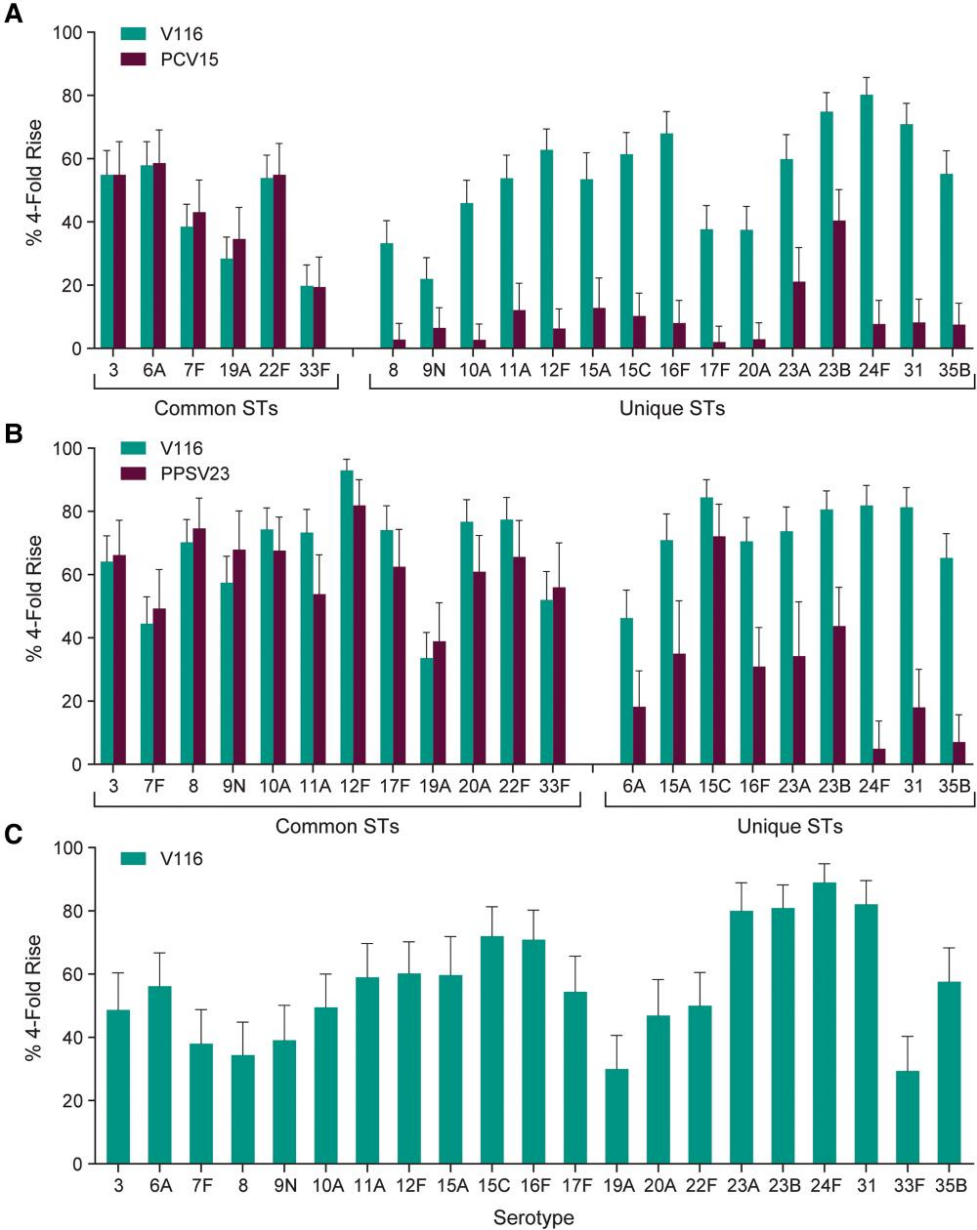
Proportions of participants with a ≥4-fold rise in serotype-specific opsonophagocytic activity geometric mean titers from prevaccination to 30 days following vaccination. Proportions and 95% confidence intervals are shown for each serotype by cohort (*A*, cohort 1; *B*, cohort 2; *C*, cohort 3), with serotypes shared in both vaccines or unique to V116 shown on the x-axis. Abbreviations: PCV15, 15-valent pneumococcal conjugate vaccine; PPSV23, 23-valent pneumococcal polysaccharide vaccine; ST, serotype; V116, 21-valent pneumococcal conjugate vaccine.

At 30 days postvaccination, serotype-specific OPA GMTs were generally comparable for all shared serotypes between V116 and PCV15 (cohort 1) or V116 and PPSV23 (cohort 2) and higher for serotypes unique to V116 (Figure 2 and Supplementary Table 3). V116 was immunogenic for all vaccine serotypes in cohort 3 (Figure 2 and Supplementary Table 3). OPA GMFRs and the proportions of participants with a ≥4-fold rise in serotype-specific OPA responses from baseline to 30 days postvaccination were consistent with OPA GMTs for all cohorts (Figure 3 and Supplementary Tables 4–6). Serotype-specific IgG GMCs were generally comparable for all shared serotypes and higher for serotypes unique to V116 in cohorts 1 and 2, and IgG GMC GMFRs and the proportions of participants with a ≥4-fold rise in GMCs from baseline to 30 days postvaccination were consistent across all cohorts (Supplementary Figure 1 and Supplementary Tables 7–10).

Serotype-specific OPA GMTs were stratified by age (50–64 years, ≥65 years) or time since prior vaccination (1–4 years, 5–9 years, ≥10 years). OPA GMTs in participants 50–64 years of age were higher for the majority of V116 serotypes compared to those ≥65 years of age following all study vaccines across cohorts (Supplementary Tables 11–13). Serotype-specific OPA GMTs by time since prior pneumococcal vaccination were generally consistent with the overall population for all study vaccines and cohorts (Supplementary Figures 2–4).

### Safety

**Figure 4.**
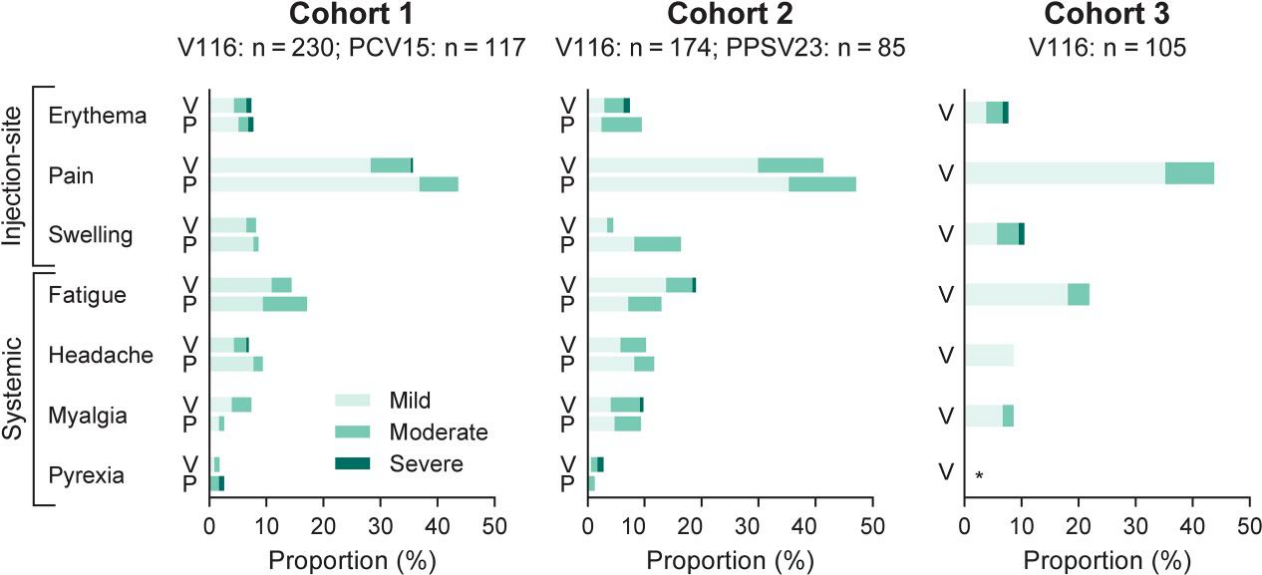
Solicited adverse events (AEs) by severity. The proportions of participants with solicited injection-site and systemic AEs by severity for each cohort are shown. For cohort 1, V = V116 (21-valent pneumococcal conjugate vaccine), P = 15-valent pneumococcal conjugate vaccine (PCV15); for cohort 2, V = V116, P = 23-valent pneumococcal polysaccharide vaccine (PPSV23); for cohort 3, V = V116. *No pyrexia occurred in cohort 3.

Across all cohorts and vaccination groups, the majority of participants experienced 1 or more AEs during the study (Table 2). The proportions of participants with AEs, solicited AEs, vaccine-related AEs, and SAEs were generally comparable between groups and across cohorts (Table 2). The most common solicited AEs following V116 were injection-site pain and fatigue. The proportions of participants with individual solicited injection-site and systemic AEs were generally comparable across groups in cohorts 1 and 2 except for injection-site swelling, which was higher following PPSV23 in cohort 2 compared to V116 (Figure 4). The majority of AEs were assessed to be a maximum intensity of mild to moderate and were of short duration (≤3 days) (Figure 4 and Supplementary Table 14). Safety data were generally consistent with the overall participant population when analyzed by sex, race, ethnicity, age, and time since last pneumococcal vaccination (data not shown).

**Table 2.** Safety Summary

Adverse Events	Cohort 1				Cohort 2				Cohort 3	
	V116 (n = 230)	95% CI	PCV15 (n = 117)	95% CI	V116 (n = 174)	95% CI	PPSV23 (n = 85)	95% CI	V116 (n = 105)	95% CI
≥1 AEs	118 (51.3)	44.6–57.9	75 (64.1)	54.7–72.8	92 (52.9)	45.2–60.5	56 (65.9)	54.8–75.8	55 (52.4)	42.4–62.2
Injection site	93 (40.4)		56 (47.9)		75 (43.1)		46 (54.1)		46 (43.8)	
Systemic	69 (30.0)		44 (37.6)		56 (32.2)		33 (38.8)		33 (31.4)	
Vaccine-related AEs^a^	107 (46.5)	39.9–53.2	66 (56.4)	46.9–65.6	87 (50.0)	42.3–57.7	52 (61.2)	50.0–71.6	51 (48.6)	38.7–58.5
Injection site	93 (40.4)		56 (47.9)		75 (43.1)		46 (54.1)		46 (43.8)	
Systemic	50 (21.7)		27 (23.1)		46 (26.4)		21 (24.7)		26 (24.8)	
Solicited injection-site AEs	92 (40.0)	…	56 (47.9)	…	75 (43.1)	…	46 (54.1)	…	46 (43.8)	…
Erythema	17 (7.4)		9 (7.7)		13 (7.5)		8 (9.4)		8 (7.6)	
Pain	82 (35.7)		51 (43.6)		72 (41.4)		40 (47.1)		46 (43.8)	
Swelling	19 (8.3)		10 (8.5)		8 (4.6)		14 (16.5)		11 (10.5)	
Solicited systemic AEs	48 (20.9)	…	25 (21.4)	…	45 (25.9)	…	20 (23.5)	…	26 (24.8)	…
Fatigue	33 (14.3)		20 (17.1)		33 (19.0)		11 (12.9)		23 (21.9)	
Headache	16 (7.0)		11 (9.4)		18 (10.3)		10 (11.8)		9 (8.6)	
Myalgia	17 (7.4)		3 (2.6)		17 (9.8)		8 (9.4)		9 (8.6)	
Pyrexia	4 (1.7)		3 (2.6)		5 (2.9)		1 (1.2)		0 (0.0)	
Serious AEs	2 (0.9)	0.1–3.1	4 (3.4)	0.9–8.5	2 (1.1)	0.1–4.1	3 (3.5)	0.7–10.0	2 (1.9)	0.2–6.7
Serious vaccine-related AEs^a^	1 (0.4)	0.0–2.4	0 (0.0)	0.0–3.1	0 (0.0)	0.0–2.1	0 (0.0)	0.0–4.2	0 (0.0)	0.0–3.5
Deaths	0 (0.0)	0.0–1.6	0 (0.0)	0.0–3.1	0 (0.0)	0.0–2.1	0 (0.0)	0.0–4.2	0 (0.0)	0.0–3.5

Abbreviations: AE, adverse event; CI, confidence interval; PCV15, 15-valent pneumococcal conjugate vaccine; PPSV23, 23-valent pneumococcal polysaccharide vaccine; V116, 21-valent pneumococcal conjugate vaccine.

^a^Determined by the investigator to be related to the vaccine.

Overall, SAEs were experienced by 1.8% of all participants (n = 13), including 1.2% of those who received V116 (n = 6), 3.4% of those who received PCV15 (n = 4), and 3.5% of those who received PPSV23 (n = 3) (Supplementary Table 15). One SAE of cellulitis at the injection site was considered related to the study vaccine in cohort 1 following V116. It occurred 6 days following vaccination and resolved following treatment 10 days later. No deaths occurred during the study. For all immunogenicity and safety readouts, data are descriptive; and, therefore, no statistical testing was performed within or between cohorts.

## DISCUSSION

This study was conducted as part of the V116 phase 3 clinical program to evaluate V116 in adults ≥50 years of age who received prior pneumococcal vaccination. Pneumococcal vaccine recommendations for adults [13] have changed to reflect accumulating data on the positive impact of vaccination and newly licensed vaccines. Thus, there is a growing population of adults who have received a variety of pneumococcal vaccines in recent years with varying serotype coverage, including PPSV23, PCV13, PCV15, and PCV20. In the US, approximately two-thirds of adults ≥65 years of age have received at least 1 pneumococcal vaccine over the years 2008–2021 [26]. Thus, there is a large and growing population of adults who have received at least 1 dose of a pneumococcal vaccine. This underscores the rationale for evaluating the pneumococcal vaccine–experienced population in this study. Study results show that regardless of pneumococcal vaccination history or time since last pneumococcal vaccination, vaccination with V116 induced immune responses to all 21 serotypes contained in the vaccine. Functional anti-PnPs antibodies were elicited to all serotypes, as evidenced by OPA responses following vaccination. This was further supported by serotype-specific IgG GMCs, as well as IgG and OPA GMFR and the proportions of participants with a ≥4-fold rise in antibody levels to each serotype. V116 immunogenicity was comparable to PCV15 and PPSV23 in cohorts 1 and 2, respectively, for shared serotypes and higher for serotypes unique to V116. The inclusion of participants with prespecified medical history conditions associated with increased risk of PD (47% of the participant population), as well as the majority of participants in the study being ≥65 years of age, demonstrates that V116 is well tolerated and immunogenic in older adults and those with a variety of preexisting conditions. These data suggest that V116 has the potential to address the majority of current residual PD in adults, including those at increased risk of IPD [27].

This study provided the opportunity to evaluate the safety and tolerability of V116 compared to 2 currently licensed vaccines, PCV15 and PPSV23. V116 was well tolerated, with a safety profile comparable to the other study vaccines. Although the study was not powered to statistically compare treatment groups, the proportions of participants with 1 or more AEs, injection-site AEs, vaccine-related AEs, and SAEs were all lower following V116. In addition, the proportions of participants with each individual solicited AE following V116 were comparable with PCV15 (cohort 1) or PPSV23 (cohort 2). One SAE assessed to be related to V116 occurred during the study. A limitation of the study is that cohort 3 was unblinded to the study intervention, which could have impacted safety reporting for these participants. Overall, V116 has a safety profile generally comparable to currently licensed pneumococcal vaccines [28–31].

The serotype composition of V116 was selected to target the burden of PD in adults and accounts for approximately 85% of IPD in US adults aged ≥65 years, which reflects the inclusion of key serotypes present in currently licensed vaccines that contribute to disease burden [15]. Importantly, V116 includes 8 serotypes that are not in any other licensed vaccine, and the additional PD coverage expected from these serotypes is approximately 30% for US adults aged ≥65 years (as described above). Specifically compared to pneumococcal vaccines licensed for use in adults, V116 covers an additional 32% of IPD compared to PCV20, and 25% of IPD compared to PPSV23 [32–35]. With this incremental increase in coverage, V116 represents a substantial benefit for adults, regardless of prior pneumococcal vaccination received. In addition, this approach is designed to afford complementary coverage in adults, combining indirect protection from infant/child vaccination programs with direct adult immunization for prevention of prevalent disease-causing serotypes. Early modeling studies suggest cost-effectiveness of V116 in addition to increased potential coverage for PD [36].

Taken together, data from this study demonstrate that V116 is well tolerated and is immunogenic for all 21 serotypes contained in the vaccine in pneumococcal vaccine–experienced adults ≥50 years of age. V116 has the potential to address an unmet medical need in adults.

## Supplementary Data

Supplementary materials are available at *Clinical Infectious Diseases* online. Consisting of data provided by the authors to benefit the reader, the posted materials are not copyedited and are the sole responsibility of the authors, so questions or comments should be addressed to the corresponding author.

## Supplementary Material

ciae383_Supplementary_Data

## Notes

***Author Contributions.*** P. S. and H. L. P. substantially contributed to the conception, design, or planning of the study, the acquisition or analysis of the data, and/or the interpretation of the results and drafting of the manuscript. J. H. C., H. S., M. D., M. H., D. G., C. G. G., W. A. O., T. Z., J. L., D. F., and N. G. substantially contributed to the conception, design, or planning of the study, the acquisition or analysis of the data, and/or the interpretation of the results. All authors critically reviewed or revised the manuscript for important intellectual content and approved the final version.

***Acknowledgments.*** The authors thank the participants and their families, study staff, and investigators in the V116-006 study group for their contributions. Medical writing support was provided by Timothy J. Chapman and editorial support was provided by Karyn Davis (both of MSD). A full list of study investigators can be found in Supplementary Table 16.

***Data sharing.*** The data sharing policy, including restrictions, of MSD is available at http://engagezone.msd.com/ds_documentation.php. Requests for access to the clinical study data can be submitted through the Engage Zone site or via email to the Data Access mailbox.

***Financial support*.** This work was supported by Merck Sharp & Dohme LLC, a subsidiary of Merck & Co., Inc., Rahway, NJ, USA (MSD).
