# Supplementary material for: A Phase 3 Clinical Study to Evaluate the Safety, Tolerability, and Immunogenicity of V116 in Pneumococcal Vaccine–Experienced Adults 50 Years of Age or Older (STRIDE-6)
Source: Clin Infect Dis. 2024 Jul 31;79(6):1366–74. doi: 10.1093/cid/ciae383 (PMC11650886; doi:10.1093/cid/ciae383)
Supplement: ciae383_Supplementary_Data [file ciae383_supplementary_data.docx]

**Supplemental material for:**

**A PHASE 3 CLINICAL STUDY TO EVALUATE THE SAFETY, TOLERABILITY, AND IMMUNOGENICITY OF V116 IN PNEUMOCOCCAL VACCINE-EXPERIENCED ADULTS 50 YEARS OF AGE OR OLDER (STRIDE-6)**

Paul Scott, MD^1*^, Miwa Haranaka, MD^2^, Jung Hyun Choi, MD^3^, Helen Stacey, MD, MPH^4^, Marc Dionne, MD^5^, David Greenberg, MD^6^, Carlos G. Grijalva, MD, MPH^7^, Walter A. Orenstein, MD^8^, Doreen Fernsler, BS^1^, Nancy Gallagher, BS^1^, Tiantian Zeng, PhD^1^, Jianing Li, PhD^1^, Heather L. Platt, MD^1^ on behalf of the STRIDE-6 study group

^1^Merck & Co., Inc., Rahway, NJ, USA; ^2^SOUSEIKAI PS Clinic, Fukuoka, Japan; ^3^Catholic University of Korea, Seoul, South Korea; ^4^Diablo Clinical Research, Walnut Creek, CA, USA; ^5^Universite Laval, Quebec, Canada; ^6^Soroka University Medical Center, Beer-Sheva, Israel; ^7^Vanderbilt University Medical Center, Nashville, TN, USA; ^8^Emory University, Atlanta, GA, USA

Supplemental Table 1: Participant enrollment by study location.

| Study Site | Participants Randomized |
| --- | --- |
| **Canada** | 75 |
| - 5 sites |  |
| **France** | 4 |
| - 2 sites |  |
| **Israel** | 99 |
| - 7 sites |  |
| **Italy** | 33 |
| - 4 sites |  |
| **Japan** | 63 |
| - 2 sites |  |
| **South Korea** | 100 |
| - 6 sites |  |
| **Spain** | 51 |
| - 5 sites |  |
| **Taiwan** | 67 |
| - 2 sites |  |
| **United States** | 225 |
| - 15 sites |  |
| **Total** | **717** |

Supplemental Table 2: Participants with pre-specified medical history conditions associated with increased risk of pneumococcal disease.

|  | **Cohort 1 (n=348)** | | **Cohort 2 (n=259)** | | **Cohort 3 (n=105)** |
| --- | --- | --- | --- | --- | --- |
| **Participants by risk factors** | V116 (n=229) | PCV15 (n=119) | V116 (n=174) | PPSV23 (n=85) | V116 (n=105) |
| Alcoholism | 1 (0.4) | 0 (0.0) | 0 (0.0) | 0 (0.0) | 0 (0.0) |
| Chronic Heart Disease | 7 (3.1) | 4 (3.4) | 4 (2.3) | 1 (1.2) | 2 (1.9) |
| Chronic Kidney Disease | 8 (3.5) | 5 (4.2) | 4 (2.3) | 1 (1.2) | 6 (5.7) |
| Chronic Liver Disease | 12 (5.2) | 6 (5.0) | 15 (8.6) | 6 (7.1) | 3 (2.9) |
| Chronic Lung Disease | 31 (13.5) | 24 (20.2) | 19 (10.9) | 14 (16.5) | 17 (16.2) |
| Diabetes | 62 (27.1) | 37 (31.1) | 37 (21.3) | 20 (23.5) | 28 (26.7) |
| Smoking | 18 (7.9) | 13 (10.9) | 13 (7.5) | 6 (7.1) | 8 (7.6) |

Supplemental Table 3: Serotype-specific OPA GMTs at day 30.

| **Cohort 1 - observed GMT (95% CI)** | | | **Cohort 2 - observed GMT (95% CI)** | | | **Cohort 3 - observed GMT (95% CI)** | |
| --- | --- | --- | --- | --- | --- | --- | --- |
| Serotype | V116 (N=229) | PCV15 (N=119) | Serotype | V116 (N=174) | PPSV23 (N=85) | Serotype | V116 (N=105) |
| **3** | 262.1 (224.0, 306.8) | 226.3 (182.0, 281.4) | **3** | 391.1 (332.8, 459.6) | 583.1 (453.5, 749.6) | 3 | 318.3 (250.0, 405.3) |
| **6A** | 1653.5 (1347.2, 2029.4) | 2076.1 (1571.4, 2742.8) | 6A | 3624.0 (3099.2, 4237.7) | 1812.3 (1226.6, 2677.6) | 6A | 2097.3 (1693.4, 2597.6) |
| **7F** | 2184.4 (1891.4, 2522.8) | 1750.3 (1404.7, 2181.0) | **7F** | 3129.8 (2609.9, 3753.3) | 4057.0 (3211.2, 5125.6) | 7F | 2051.3 (1630.2, 2581.0) |
| 8 | 1273.0 (1115.1, 1453.3) | 345.8 (250.5, 477.5) | **8** | 2320.1 (1987.3, 2708.7) | 2723.2 (2197.4, 3374.8) | 8 | 1486.8 (1230.5, 1796.6) |
| 9N | 3805.1 (3324.0, 4356.0) | 2176.5 (1809.6, 2617.9) | **9N** | 7214.4 (6062.9, 8584.6) | 6482.5 (4908.9, 8560.7) | 9N | 4054.5 (3389.4, 4850.2) |
| 10A | 1986.2 (1637.7, 2408.9) | 467.5 (337.0, 648.5) | **10A** | 3976.8 (3360.7, 4705.8) | 1797.6 (1136.2, 2843.9) | 10A | 2564.0 (1959.1, 3355.6) |
| 11A | 1998.5 (1696.9, 2353.8) | 335.6 (228.9, 491.8) | **11A** | 2846.6 (2411.0, 3360.8) | 1736.6 (1367.1, 2206.0) | 11A | 2373.0 (1905.4, 2955.4) |
| 12F | 981.8 (782.4, 1232.1) | 80.5 (54.0, 120.1) | **12F** | 2552.6 (2120.5,3072.9) | 1402.5 (912.2, 2156.4) | 12F | 1235.3 (948.3, 1609.2) |
| 15A | 4184.9 (3548.3, 4935.6) | 877.2 (616.2, 1248.7) | 15A | 6185.2 (5179.3, 7386.6) | 1668.2 (1234.4,2254.5) | 15A | 4328.6 (3378.7, 5545.7) |
| 15C | 2307.8 (1878.4, 2835.4) | 539.6 (371.1, 784.6) | 15C | 4334.4 (3563.8, 5271.5) | 1470.4 (978.6, 2209.3) | 15C | 2191.9 (1573.2, 3053.9) |
| 16F | 3060.5 (2633.8, 3556.3) | 392.3 (301.3, 510.8) | 16F | 4626.5 (3861.8, 5542.6) | 832.8 (604.3, 1147.6) | 16F | 2477.0 (1887.2, 3251.2) |
| 17F | 3599.8 (3134.5, 4134.3) | 939.6 (693.7, 1272.6) | **17F** | 5963.8 (5036.6, 7061.7) | 4367.3 (3372.5, 5655.7) | 17F | 3836.7 (3063.4, 4805.1) |
| **19A** | 1513.8 (1318.4, 1738.1) | 2022.9 (1634.1, 2504.3) | **19A** | 2528.9 (2201.7, 2904.9) | 3241.5 (2646.0, 3971.0) | 19A | 1533.8 (1272.4, 1848.9) |
| 20A | 2847.4 (2433.3, 3331.8) | 1058.9 (829.9, 1351.1) | **20A** | 6005.5 (4919.8, 7330.8) | 3393.9 (2536.9, 4540.5) | 20A | 2433.4 (1880.5, 3148.9) |
| **22F** | 1983.8 (1698.3, 2317.4) | 1595.6 (1227.1, 2074.6) | **22F** | 4389.2 (3541.1, 5440.3) | 2524.0 (1834.5, 3472.5) | 22F | 1913.5 (1453.5, 2519.0) |
| 23A | 2363.9 (1857.4, 3008.5) | 310.2 (202.1, 476.0) | 23A | 4253.4 (3417.6, 5293.5) | 433.6 (247.5, 759.5) | 23A | 3967.2 (2764.8, 5692.7) |
| 23B | 673.2 (517.1, 876.4) | 153.0 (98.7, 237.1) | 23B | 1530.7 (1196.5, 1958.3) | 203.9 (127.6, 325.6) | 23B | 844.0 (608.2, 1171.4) |
| 24F | 1822.6 (1411.6, 2353.3) | 106.6 (69.7, 162.9) | 24F | 2746.1 (2257.9, 3339.9) | 48.5 (28.6, 82.1) | 24F | 2041.5 (1500.8, 2777.1) |
| 31 | 3018.4 (2473.6, 3683.3) | 113.2 (74.5, 172.1) | 31 | 4413.5 (3530.2, 5517.7) | 171.8 (99.9, 295.6) | 31 | 3285.5 (2485.0, 4343.8) |
| **33F** | 4311.9 (3625.1,5128.9) | 3397.2 (2665.3, 4330.0) | **33F** | 8162.9 (6407.2, 10399.7) | 8761.9 (6157.4, 12468.1) | 33F | 4654.3 (3532.1, 6133.1) |
| 35B | 6703.1 (5732.7, 7837.8) | 1019.1 (739.9, 1403.7) | 35B | 8143.5 (6761.4, 9808.1) | 1527.7 (1169.5, 1995.5) | 35B | 5836.8 (4693.6, 7258.6) |

- Bold denotes pneumococcal serotypes shared between the two vaccines within a given Cohort

- Number of participants contributing to the analysis: Cohort 1 - n=175-212 for V116 group, n=91-114 for PCV15 group; Cohort 2 - n=125-161 for V116 group, n=58-76 for PPSV23 group; Cohort 3 – n=82-99

- Serotype 15C represents the immune response to the deOAc15B polysaccharide as the molecular structure for deOAc15B and 15C are similar; anti-15C immune responses are assessed in this study

- CI=confidence interval; N=number of participants randomized and vaccinated

- V116 contains all serotypes listed in Table; PCV15 contains serotypes 1, 3, 4, 5, 6A, 7F, 9V, 14, 18C, 19A, 19F, 23F, 22F, and 33F; PPSV23 contains serotypes 1, 2, 3, 4, 5, 6B, 7F, 8, 9N, 9V, 10A, 11A, 12F, 14, 15B, 17F, 18C, 19A, 19F, 20, 22F, 23F, and 33F

Supplemental Table 4: Summary of serotype-specific OPA antibody responses – Cohort 1.

| **STs** |  | **V116 (N=229)** | | **PCV15 (N=119)** | |  | **V116 (N=229)** | | **PCV15 (N=119)** | |
| --- | --- | --- | --- | --- | --- | --- | --- | --- | --- | --- |
| **Common** | Endpoint | n | Response (95% CI) | n | Response (95% CI) | **Unique** | n | Response (95% CI) | n | Response (95% CI) |
| 3 | GMT (Day 1) | 191 | 48.1 (39.0, 59.4) | 103 | 46.7 (34.7, 62.8) | 15A | 187 | 699.4 (553.5, 883.8) | 91 | 765.4 (560.3, 1045.7) |
|  | GMT (Day 30) | 197 | 262.1 (224.0, 306.8) | 103 | 226.3 (182.0, 281.4) |  | 175 | 4184.9 (3548.3, 4935.6) | 93 | 877.2 (616.2, 1248.7) |
|  | GMFR | 164 | 4.4 (3.7, 5.3) | 91 | 4.0 (3.1, 5.3) |  | 142 | 5.6 (4.3, 7.4) | 78 | 1.2 (0.9, 1.5) |
|  | % ≥4-fold rise | 164 | 54.9% (46.9, 62.6) | 91 | 54.9% (50/91) (44.2, 65.4) |  | 142 | 53.5% (45.0, 61.9) | 78 | 12.8% (10/78) (6.3, 22.3) |
| 6A | GMT (Day 1) | 202 | 226.1 (175.3, 291.6) | 108 | 255.8 (175.9, 371.9) | 15C | 217 | 251.0 (194.7, 323.5) | 113 | 375.1 (257.1, 547.3) |
|  | GMT (Day 30) | 191 | 1653.5 (1347.2, 2029.4) | 94 | 2076.1 (1571.4, 2742.8) |  | 206 | 2307.8 (1878.4, 2835.4) | 110 | 539.6 (371.1, 784.6) |
|  | GMFR | 171 | 6.2 (5.0, 7.7) | 87 | 7.1 (4.9, 10.3) |  | 197 | 8.5 (6.7, 10.9) | 108 | 1.3 (1.0, 1.7) |
|  | % ≥4-fold rise | 171 | 57.9% (50.1, 65.4) | 87 | 58.6% (51/87) (47.6, 69.1) |  | 197 | 61.4% (54.2, 68.3) | 108 | 10.2% (11/108) (5.2, 17.5) |
| 7F | GMT (Day 1) | 216 | 476.3 (374.4, 605.9) | 107 | 463.5 (337.4, 636.8) | 16F | 206 | 342.7 (284.8, 412.3) | 108 | 377.3 (290.7, 489.7) |
|  | GMT (Day 30) | 209 | 2184.4 (1891.4, 2522.8) | 110 | 1750.3 (1404.7, 2181.0) |  | 187 | 3060.5 (2633.8, 3556.3) | 107 | 392.3 (301.3, 510.8) |
|  | GMFR | 200 | 4.1 (3.3, 5.1) | 102 | 3.9 (2.8, 5.4) |  | 172 | 7.1 (5.9, 8.5) | 100 | 1.1 (0.9, 1.3) |
|  | % ≥4-fold rise | 200 | 38.5% (31.7, 45.6) | 102 | 43.1% (44/102) (33.4, 53.3) |  | 172 | 68.0% (60.5, 74.9) | 100 | 8.0% (8/100) (3.5, 15.2) |
| 19A | GMT (Day 1) | 216 | 607.5 (497.7, 741.6) | 110 | 596.5 (450.5, 789.7) | 17F | 212 | 984.2 (810.1, 1195.8) | 108 | 1041.5 (779.8, 1391.0) |
|  | GMT (Day 30) | 204 | 1513.8 (1318.4, 1738.1) | 109 | 2022.9 (1634.1, 2504.3) |  | 194 | 3599.8 (3134.5, 4134.3) | 108 | 939.6 (693.7, 1272.6) |
|  | GMFR | 194 | 2.4 (2.0, 2.8) | 104 | 3.3 (2.5, 4.4) |  | 183 | 3.7 (3.0, 4.5) | 101 | 0.9 (0.8, 1.1) |
|  | % ≥4-fold rise | 194 | 28.4% (22.1, 35.2) | 104 | 34.6% (36/104) (25.6, 44.6) |  | 183 | 37.7% (30.7, 45.2) | 101 | 2.0% (2/101) (0.2, 7.0) |
| 22F | GMT (Day 1) | 212 | 270.4 (206.8, 353.4) | 109 | 194.9 (129.0, 294.5) | 20A | 212 | 786.4 (648.0, 954.3) | 110 | 1145.3 (918.1, 1428.7) |
|  | GMT (Day 30) | 206 | 1983.8 (1698.3, 2317.4) | 108 | 1595.6 (1227.1, 2074.6) |  | 195 | 2847.4 (2433.3, 3331.8) | 110 | 1058.9 (829.9, 1351.1) |
|  | GMFR | 193 | 7.0 (5.5, 9.0) | 102 | 7.1 (4.8, 10.6) |  | 184 | 3.7 (3.0, 4.5) | 105 | 0.9 (0.8, 1.0) |
|  | % ≥4-fold rise | 193 | 53.9% (46.6, 61.1) | 102 | 54.9% (56/102) (44.7, 64.8) |  | 184 | 37.5% (30.5, 44.9) | 105 | 2.9% (3/105) (0.6, 8.1) |
| 33F | GMT (Day 1) | 210 | 2002.1 (1702.4, 2354.4) | 108 | 2080.9 (1646.5, 2630.0) | 23A | 179 | 227.2 (166.4, 310.3) | 96 | 194.4 (128.7, 293.8) |
|  | GMT (Day 30) | 188 | 4311.9 (3625.1, 5128.9) | 99 | 3397.2 (2665.3, 4330.0) |  | 202 | 2363.9 (1857.4, 3008.5) | 91 | 310.2 (202.1, 476.0) |
|  | GMFR | 177 | 2.2 (1.8, 2.6) | 93 | 1.7 (1.3, 2.4) |  | 157 | 8.4 (6.2, 11.6) | 76 | 1.4 (1.0, 1.9) |
|  | % ≥4-fold rise | 177 | 19.8% (14.2, 26.4) | 93 | 19.4% (18/93) (11.9, 28.9) |  | 157 | 59.9% (51.8, 67.6) | 76 | 21.1% (16/76) (12.5, 31.9) |
| **Unique** |  |  |  |  |  |  |  |  |  |  |
| 8 | GMT (Day 1) | 216 | 443.9 (369.3, 533.6) | 110 | 396.9 (294.6, 534.8) | 23B | 218 | 26.6 (20.7, 34.1) | 114 | 32.2 (22.4, 46.2) |
|  | GMT (Day 30) | 208 | 1273.0 (1115.1, 1453.3) | 113 | 345.8 (250.5, 477.5) |  | 197 | 673.2 (517.1, 876.4) | 110 | 153.0 (98.7, 237.1) |
|  | GMFR | 198 | 2.8 (2.4, 3.4) | 108 | 0.9 (0.7, 1.0) |  | 191 | 21.4 (16.2, 28.1) | 109 | 4.2 (2.9, 6.0) |
|  | % ≥4-fold rise | 198 | 33.3% (26.8, 40.4) | 108 | 2.8% (3/108) (0.6, 7.9) |  | 191 | 74.9% (68.1, 80.9) | 109 | 40.4% (44/109) (31.1, 50.2) |
| 9N | GMT (Day 1) | 219 | 1661.7 (1419.0, 1945.9) | 111 | 1596.0 (1285.7, 1981.2) | 24F | 209 | 50.3 (37.9, 66.7) | 107 | 100.4 (68.2, 147.8) |
|  | GMT (Day 30) | 191 | 3805.1 (3324.0, 4356.0) | 111 | 2176.5 (1809.6, 2617.9) |  | 201 | 1822.6 (1411.6, 2353.3) | 97 | 106.6 (69.7, 162.9) |
|  | GMFR | 186 | 2.3 (2.0, 2.7) | 108 | 1.4 (1.1, 1.7) |  | 187 | 25.8 (19.5, 34.0) | 91 | 1.0 (0.8, 1.3) |
|  | % ≥4-fold rise | 186 | 22.0% (16.3, 28.7) | 108 | 6.5% (7/108) (2.6, 12.9) |  | 187 | 80.2% (73.8, 85.7) | 91 | 7.7% (7/91) (3.1, 15.2) |
| 10A | GMT (Day 1) | 215 | 421.8 (323.1, 550.6) | 114 | 446.2 (324.9, 612.7) | 31 | 210 | 151.8 (114.5, 201.1) | 104 | 127.0 (84.5, 190.8) |
|  | GMT (Day 30) | 209 | 1986.2 (1637.7, 2408.9) | 112 | 467.5 (337.0, 648.5) |  | 194 | 3018.4 (2473.6, 3683.3) | 108 | 113.2 (74.5, 172.1) |
|  | GMFR | 200 | 4.3 (3.5, 5.4) | 111 | 1.0 (0.9, 1.2) |  | 179 | 16.7 (12.5, 22.3) | 97 | 0.9 (0.7, 1.2) |
|  | % ≥4-fold rise | 200 | 46.0% (38.9, 53.2) | 111 | 2.7% (3/111) (0.6, 7.7) |  | 179 | 70.9% (63.7, 77.5) | 97 | 8.2% (8/97) (3.6, 15.6) |
| 11A | GMT (Day 1) | 210 | 261.1 (202.9, 335.9) | 104 | 289.1 (194.7, 429.2) | 35B | 213 | 1159.2 (959.7, 1400.3) | 114 | 976.4 (717.3, 1329.1) |
|  | GMT (Day 30) | 197 | 1998.5 (1696.9, 2353.8) | 100 | 335.6 (228.9, 491.8) |  | 194 | 6703.1 (5732.7, 7837.8) | 107 | 1019.1 (739.9, 1403.7) |
|  | GMFR | 186 | 6.5 (5.2, 8.2) | 91 | 1.2 (0.9, 1.7) |  | 183 | 6.0 (4.8, 7.4) | 106 | 1.0 (0.8, 1.2) |
|  | % ≥4-fold rise | 186 | 53.8% (46.3, 61.1) | 91 | 12.1% (11/91) (6.2, 20.6) |  | 183 | 55.2% (47.7, 62.5) | 106 | 7.5% (8/106) (3.3, 14.3) |
| 12F | GMT (Day 1) | 220 | 57.1 (42.9, 75.9) | 113 | 72.3 (48.5, 107.7) |  |  |  |  |  |
|  | GMT (Day 30) | 212 | 981.8 (782.4, 1232.1) | 114 | 80.5 (54.0, 120.1) |  |  |  |  |  |
|  | GMFR | 207 | 11.8 (9.0, 15.4) | 112 | 1.1 (1.0, 1.3) |  |  |  |  |  |
|  | % ≥4-fold rise | 207 | 62.8% (55.8, 69.4) | 112 | 6.3% (7/112) (2.5, 12.5) |  |  |  |  |  |

Supplemental Table 5: Summary of serotype-specific OPA antibody responses – Cohort 2.

| **STs** |  | **V116 (N=174)** | | **PPSV23 (N=85)** | |  | **V116 (N=174)** | | **PPSV23 (N=85)** | |
| --- | --- | --- | --- | --- | --- | --- | --- | --- | --- | --- |
| **Common** | Endpoint | n | Response (95% CI) | n | Response (95% CI) | **Unique** | n | Response (95% CI) | n | Response (95% CI) |
| 3 | GMT (Day 1) | 149 | 56.5 (45.1, 70.8) | 77 | 68.1 (48.8, 95.1) | 6A | 150 | 798.5 (610.3, 1044.7) | 76 | 978.3 (656.6, 1457.6) |
|  | GMT (Day 30) | 149 | 391.1 (332.8, 459.6) | 75 | 583.1 (453.5, 749.6) |  | 152 | 3624.0 (3099.2, 4237.7) | 74 | 1812.3 (1226.6, 2677.6) |
|  | GMFR | 131 | 6.2 (5.0, 7.6) | 68 | 7.5 (5.3, 10.5) |  | 136 | 4.2 (3.3, 5.3) | 66 | 1.8 (1.4, 2.4) |
|  | % ≥4-fold rise | 131 | 64.1% (55.3, 72.3) | 68 | 66.2% (53.7, 77.2) |  | 136 | 46.3% (37.7, 55.1) | 66 | 18.2% (9.8, 29.6) |
| 7F | GMT (Day 1) | 164 | 675.0 (524.2, 869.3) | 82 | 992.1 (731.7, 1345.1) | 15A | 134 | 590.4 (453.3, 768.9) | 56 | 465.9 (300.1, 723.4) |
|  | GMT (Day 30) | 150 | 3129.8 (2609.9, 3753.3) | 70 | 4057.0 (3211.2, 5125.6) |  | 134 | 6185.2 (5179.3, 7386.6) | 63 | 1668.2 (1234.4, 2254.5) |
|  | GMFR | 146 | 4.6 (3.6, 5.9) | 69 | 4.2 (3.1, 5.8) |  | 110 | 9.0 (6.8, 11.8) | 40 | 3.0 (2.0, 4.5) |
|  | % ≥4-fold rise | 146 | 44.5% (36.3, 53.0) | 69 | 49.3% (37.0, 61.6) |  | 110 | 70.9% (61.5, 79.2) | 40 | 35.0% (20.6, 51.7) |
| 8 | GMT (Day 1) | 161 | 116.8 (84.7, 161.0) | 79 | 118.3 (74.0, 189.2) | 15C | 157 | 120.8 (86.9, 168.0) | 79 | 84.5 (55.9, 127.7) |
|  | GMT (Day 30) | 161 | 2320.1 (1987.3, 2708.7) | 75 | 2723.2 (2197.4, 3374.8) |  | 152 | 4334.4 (3563.8, 5271.5) | 72 | 1470.4 (978.6, 2209.3) |
|  | GMFR | 151 | 16.6 (12.2, 22.7) | 71 | 18.6 (11.9, 29.1) |  | 141 | 30.6 (22.5, 41.5) | 68 | 12.7 (8.5, 18.8) |
|  | % ≥4-fold rise | 151 | 70.2% (62.2, 77.4) | 71 | 74.6% (62.9, 84.2) |  | 141 | 84.4% (77.3, 90.0) | 68 | 72.1% (59.9, 82.3) |
| 9N | GMT (Day 1) | 163 | 1121.9 (918.8, 1369.8) | 77 | 1106.0 (798.4, 1532.3) | 16F | 156 | 419.5 (336.1, 523.6) | 75 | 397.2 (283.4, 556.6) |
|  | GMT (Day 30) | 143 | 7214.4 (6062.9, 8584.6) | 58 | 6482.5 (4908.9, 8560.7) |  | 146 | 4626.5 (3861.8, 5542.6) | 74 | 832.8 (604.3, 1147.6) |
|  | GMFR | 136 | 5.9 (4.7, 7.4) | 53 | 6.8 (4.7, 9.8) |  | 132 | 9.8 (7.8, 12.4) | 68 | 2.0 (1.5, 2.7) |
|  | % ≥4-fold rise | 136 | 57.4% (48.6, 65.8) | 53 | 67.9% (53.7, 80.1) |  | 132 | 70.5% (61.9, 78.1) | 68 | 30.9% (20.2, 43.3) |
| 10A | GMT (Day 1) | 163 | 206.3 (145.9, 291.7) | 81 | 151.7 (89.8, 256.3) | 23A | 128 | 182.8 (125.5, 266.3) | 56 | 171.5 (93.7, 314.0) |
|  | GMT (Day 30) | 155 | 3976.8 (3360.7, 4705.8) | 73 | 1797.6 (1136.2, 2843.9) |  | 156 | 4253.4 (3417.6, 5293.5) | 60 | 433.6 (247.5, 759.5) |
|  | GMFR | 148 | 17.1 (12.7, 23.0) | 71 | 11.8 (7.5, 18.6) |  | 118 | 19.0 (13.5, 26.9) | 38 | 2.5 (1.3, 4.9) |
|  | % ≥4-fold rise | 148 | 74.3% (66.5, 81.1) | 71 | 67.6% (55.5, 78.2) |  | 118 | 73.7% (64.8, 81.4) | 38 | 34.2% (19.6, 51.4) |
| 11A | GMT (Day 1) | 159 | 132.0 (91.3, 190.8) | 76 | 178 (105.1, 301.6) | 23B | 165 | 34.6 (25.3, 47.2) | 80 | 31.7 (20.1, 49.8) |
|  | GMT (Day 30) | 142 | 2846.6 (2411.0, 3360.8) | 71 | 1736.6 (1367.1, 2206.0) |  | 160 | 1530.7 (1196.5, 1958.3) | 75 | 203.9 (127.6, 325.6) |
|  | GMFR | 131 | 21.0 (14.6, 30.4) | 65 | 9.2 (5.9, 14.4) |  | 155 | 35.5 (25.9, 48.6) | 71 | 5.2 (3.5, 7.6) |
|  | % ≥4-fold rise | 131 | 73.3% (64.8, 80.6) | 65 | 53.8% (41.0, 66.3) |  | 155 | 80.6% (73.5, 86.5) | 71 | 43.7% (31.9, 56.0) |
| 12F | GMT (Day 1) | 168 | 18.9 (14.8, 24.3) | 82 | 21.8 (14.7, 32.4) | 24F | 144 | 67.4 (47.1, 96.4) | 80 | 51.2 (31.8, 82.4) |
|  | GMT (Day 30) | 160 | 2552.6 (2120.5, 3072.9) | 73 | 1402.5 (912.2, 2156.4) |  | 151 | 2746.1 (2257.9, 3339.9) | 63 | 48.5 (28.6, 82.1) |
|  | GMFR | 157 | 82.2 (62.9, 107.4) | 72 | 41.9 (26.1, 67.2) |  | 127 | 31.1 (22.8, 42.6) | 61 | 0.9 (0.7, 1.3) |
|  | % ≥4-fold rise | 157 | 93.0% (87.8, 96.5) | 72 | 81.9% (71.1, 90.0) |  | 127 | 81.9% (74.1, 88.2) | 61 | 4.9% (1.0, 13.7) |
| 17F | GMT (Day 1) | 160 | 410.6 (306.3, 550.6) | 81 | 481.9 (320.6, 724.4) | 31 | 152 | 96.7 (66.9, 139.6) | 77 | 82.5 (50.2, 135.6) |
|  | GMT (Day 30) | 125 | 5963.8 (5036.6, 7061.7) | 67 | 4367.3 (3372.5, 5655.7) |  | 146 | 4413.5 (3530.2, 5517.7) | 68 | 171.8 (99.9, 295.6) |
|  | GMFR | 116 | 13.5 (9.5, 19.2) | 64 | 8.1 (5.6, 11.7) |  | 134 | 35.6 (24.8, 51.2) | 61 | 1.7 (1.1, 2.6) |
|  | % ≥4-fold rise | 116 | 74.1% (65.2, 81.8) | 64 | 62.5% (49.5, 74.3) |  | 134 | 81.3% (73.7, 87.5) | 61 | 18.0% (9.4, 30.0) |
| 19A | GMT (Day 1) | 161 | 862.6 (701.3, 1061.2) | 82 | 952.7 (707.6, 1282.7) | 35B | 165 | 1151.4 (927.4, 1429.5) | 77 | 1108.7 (788.4, 1559.1) |
|  | GMT (Day 30) | 158 | 2528.9 (2201.7, 2904.9) | 74 | 3241.5 (2646.0, 3971.0) |  | 148 | 8143.5 (6761.4, 9808.1) | 76 | 1527.7 (1169.5, 1995.5) |
|  | GMFR | 149 | 2.8 (2.3, 3.3) | 72 | 3.6 (2.7, 4.7) |  | 144 | 7.2 (5.6, 9.2) | 71 | 1.4 (1.1, 1.8) |
|  | % ≥4-fold rise | 149 | 33.6% (26.0, 41.7) | 72 | 38.9% (27.6, 51.1) |  | 144 | 65.3% (56.9, 73.0) | 71 | 7.0% (2.3, 15.7) |
| 20A | GMT (Day 1) | 157 | 552.8 (431.8, 707.6) | 78 | 535.8 (368.7, 778.6) |  |  |  |  |  |
|  | GMT (Day 30) | 138 | 6005.5 (4919.8, 7330.8) | 72 | 3393.9 (2536.9, 4540.5) |  |  |  |  |  |
|  | GMFR | 129 | 12.2 (9.3, 16.0) | 69 | 6.0 (4.4, 8.3) |  |  |  |  |  |
|  | % ≥4-fold rise | 129 | 76.7% (68.5, 83.7) | 69 | 60.9% (48.4, 72.4) |  |  |  |  |  |
| 22F | GMT (Day 1) | 148 | 137.8 (94.6, 200.7) | 76 | 116.1 (67.1, 200.8) |  |  |  |  |  |
|  | GMT (Day 30) | 143 | 4389.2 (3541.1, 5440.3) | 71 | 2524.0 (1834.5, 3472.5) |  |  |  |  |  |
|  | GMFR | 124 | 26.3 (17.7, 39.2) | 64 | 14.8 (8.7, 25.1) |  |  |  |  |  |
|  | % ≥4-fold rise | 124 | 77.4% (69.0, 84.4) | 64 | 65.6% (52.7, 77.1) |  |  |  |  |  |
| 33F | GMT (Day 1) | 158 | 1383.6 (1124.4, 1702.6) | 74 | 1210.8 (814.8, 1799.4) |  |  |  |  |  |
|  | GMT (Day 30) | 131 | 8162.9 (6407.2, 10399.7) | 59 | 8761.9 (6157.4, 12468.1) |  |  |  |  |  |
|  | GMFR | 125 | 6.3 (4.8, 8.4) | 50 | 9.2 (5.6, 15.3) |  |  |  |  |  |
|  | % ≥4-fold rise | 125 | 52.0% (42.9, 61.0) | 50 | 56.0% (41.3, 70.0) |  |  |  |  |  |

Supplemental Table 6: Summary of serotype-specific OPA antibody responses – Cohort 3.

|  |  | **V116 (N=105)** | |  | **V116 (N=105)** | |
| --- | --- | --- | --- | --- | --- | --- |
| **Serotype** | Endpoint | n | Response (95% CI) | **Serotype** | n | Response (95% CI) |
| 3 | GMT (Day 1) | 91 | 49.1 (36.6, 65.7) | 17F | 101 | 650.2 (465.1, 908.8) |
|  | GMT (Day 30) | 85 | 318.3 (250.0, 405.3) |  | 82 | 3836.7 (3063.4, 4805.1) |
|  | GMFR | 76 | 5.0 (3.6, 7.0) |  | 79 | 6.5 (4.6, 9.3) |
|  | % ≥4-fold rise | 76 | 48.7% (37.0, 60.4) |  | 79 | 54.4% (42.8, 65.7) |
| 6A | GMT (Day 1) | 99 | 398.6 (287.7, 552.3) | 19A | 100 | 628.7 (471.0, 839.2) |
|  | GMT (Day 30) | 93 | 2097.3 (1693.4, 2597.6) |  | 93 | 1533.8 (1272.4, 1848.9) |
|  | GMFR | 89 | 5.5 (4.1, 7.4) |  | 90 | 2.5 (1.8, 3.3) |
|  | % ≥4-fold rise | 89 | 56.2% (45.3, 66.7) |  | 90 | 30.0% (20.8, 40.6) |
| 7F | GMT (Day 1) | 101 | 469.0 (336.4, 653.9) | 20A | 96 | 558.5 (395.1, 789.4) |
|  | GMT (Day 30) | 96 | 2051.3 (1630.2, 2581.0) |  | 88 | 2433.4 (1880.5, 3148.9) |
|  | GMFR | 92 | 3.9 (2.9, 5.3) |  | 81 | 4.4 (3.1, 6.1) |
|  | % ≥4-fold rise | 92 | 38.0% (28.1, 48.8) |  | 81 | 46.9% (35.7, 58.3) |
| 8 | GMT (Day 1) | 103 | 349.3 (239.2, 510.0) | 22F | 98 | 218.1 (140.4, 338.8) |
|  | GMT (Day 30) | 98 | 1486.8 (1230.5, 1796.6) |  | 99 | 1913.5 (1453.5, 2519.0) |
|  | GMFR | 96 | 3.8 (2.7, 5.3) |  | 94 | 7.5 (4.9, 11.4) |
|  | % ≥4-fold rise | 96 | 34.4% (25.0, 44.8) |  | 94 | 50.0% (39.5, 60.5) |
| 9N | GMT (Day 1) | 102 | 1163.7 (898.2, 1507.8) | 23A | 77 | 206.9 (126.7, 337.6) |
|  | GMT (Day 30) | 90 | 4054.5 (3389.4, 4850.2) |  | 86 | 3967.2 (2764.8, 5692.7) |
|  | GMFR | 87 | 3.5 (2.7, 4.5) |  | 65 | 16.3 (10.3, 25.7) |
|  | % ≥4-fold rise | 87 | 39.1% (28.8, 50.1) |  | 65 | 80.0% (68.2, 88.9) |
| 10A | GMT (Day 1) | 102 | 475.4 (321.8, 702.5) | 23B | 102 | 22.2 (15.5, 31.6) |
|  | GMT (Day 30) | 96 | 2564.0 (1959.1, 3355.6) |  | 97 | 844.0 (608.2, 1171.4) |
|  | GMFR | 93 | 4.7 (3.4, 6.5) |  | 94 | 26.1 (17.5, 38.9) |
|  | % ≥4-fold rise | 93 | 49.5% (38.9, 60.0) |  | 94 | 80.9% (71.4, 88.2) |
| 11A | GMT (Day 1) | 100 | 225.6 (146.7, 346.8) | 24F | 96 | 35.8 (24.6, 52.3) |
|  | GMT (Day 30) | 87 | 2373.0 (1905.4, 2955.4) |  | 90 | 2041.5 (1500.8, 2777.1) |
|  | GMFR | 83 | 9.5 (6.2, 14.4) |  | 82 | 39.5 (27.4, 56.9) |
|  | % ≥4-fold rise | 83 | 59.0% (47.7, 69.7) |  | 82 | 89.0% (80.2, 94.9) |
| 12F | GMT (Day 1) | 98 | 64.2 (41.6, 99.0) | 31 | 97 | 89.0 (57.8, 137.0) |
|  | GMT (Day 30) | 99 | 1235.3 (948.3, 1609.2) |  | 90 | 3285.5 (2485.0, 4343.8) |
|  | GMFR | 93 | 13.7 (9.0, 20.8) |  | 84 | 30.5 (19.1, 48.6) |
|  | % ≥4-fold rise | 93 | 60.2% (49.5, 70.2) |  | 84 | 82.1% (72.3, 89.6) |
| 15A | GMT (Day 1) | 77 | 609.2 (408.6, 908.4) | 33F | 99 | 1770.5 (1323.7, 2368.2) |
|  | GMT (Day 30) | 86 | 4328.6 (3378.7, 5545.7) |  | 88 | 4654.3 (3532.1, 6133.1) |
|  | GMFR | 62 | 7.2 (4.6, 11.2) |  | 85 | 2.7 (2.0, 3.7) |
|  | % ≥4-fold rise | 62 | 59.7% (46.4, 71.9) |  | 85 | 29.4% (20.0, 40.3) |
| 15C | GMT (Day 1) | 98 | 160.4 (105.3, 244.4) | 35B | 99 | 943.7 (693.1, 1285.0) |
|  | GMT (Day 30) | 89 | 2191.9 (1573.2, 3053.9) |  | 90 | 5836.8 (4693.6, 7258.6) |
|  | GMFR | 82 | 11.6 (8.1, 16.4) |  | 85 | 5.7 (4.2, 7.9) |
|  | % ≥4-fold rise | 82 | 72.0% (60.9, 81.3) |  | 85 | 57.6% (46.4, 68.3) |
| 16F | GMT (Day 1) | 102 | 290.8 (225.5, 375.1) |  |  |  |
|  | GMT (Day 30) | 89 | 2477.0 (1887.2, 3251.2) |  |  |  |
|  | GMFR | 86 | 7.2 (5.5, 9.6) |  |  |  |
|  | % ≥4-fold rise | 86 | 70.9% (60.1, 80.2) |  |  |  |

Supplemental Figure 1: Serotype-specific IgG GMCs at day 30.


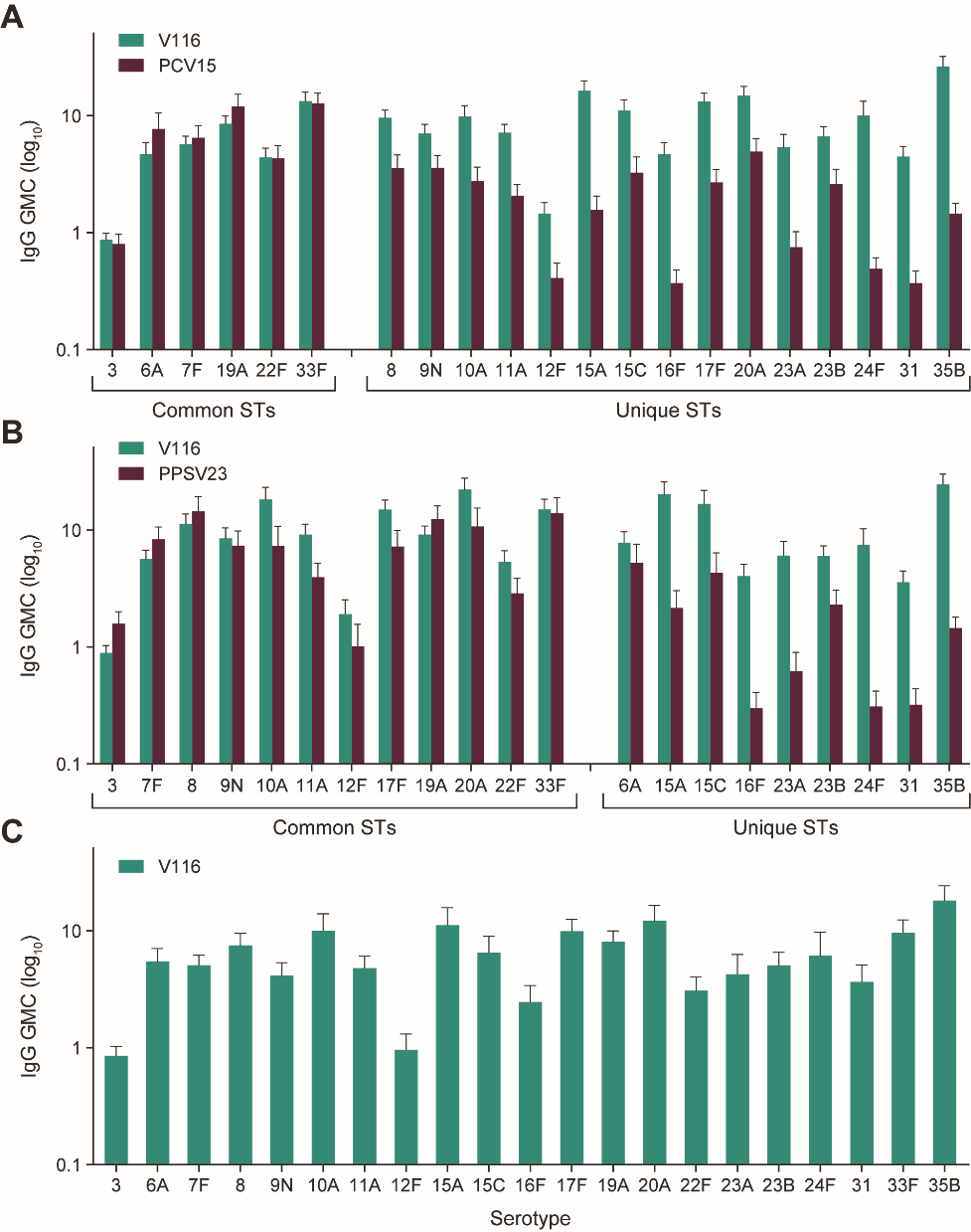


Supplemental Table 7: Serotype-specific IgG GMCs at day 30.

| **Cohort 1 - observed GMC (95% CI)** | | | **Cohort 2 - observed GMC (95% CI)** | | | **Cohort 3 - observed GMC (95% CI)** | |
| --- | --- | --- | --- | --- | --- | --- | --- |
| Serotype | V116 (N=229) | PCV15 (N=119) | Serotype | V116 (N=174) | PPSV23 (N=85) | Serotype | V116 (N=105) |
| **3** | 0.87 (0.77, 0.99) | 0.80 (0.65, 0.97) | **3** | 0.89 (0.77, 1.03) | 1.58 (1.25, 2.00) | 3 | 0.85 (0.70, 1.03) |
| **6A** | 4.69 (3.72, 5.90) | 7.69 (5.60, 10.57) | 6A | 7.81 (6.30, 9.68) | 5.24 (3.62, 7.56) | 6A | 5.48 (4.27, 7.03) |
| **7F** | 5.71 (4.89, 6.68) | 6.45 (5.08, 8.19) | **7F** | 5.65 (4.75, 6.73) | 8.34 (6.57, 10.59) | 7F | 5.07 (4.12, 6.23) |
| 8 | 9.58 (8.21, 11.17) | 3.56 (2.74, 4.62) | **8** | 11.29 (9.28, 13.73) | 14.47 (10.86, 19.27) | 8 | 7.48 (5.87, 9.53) |
| 9N | 7.06 (5.92, 8.43) | 3.59 (2.83, 4.55) | **9N** | 8.48 (6.89, 10.44) | 7.32 (5.47, 9.78) | 9N | 4.15 (3.23, 5.34) |
| 10A | 9.86 (7.99, 12.18) | 2.77 (2.12, 3.63) | **10A** | 18.21 (14.30, 23.18) | 7.29 (4.94, 10.74) | 10A | 10.03 (7.21, 13.98) |
| 11A | 7.16 (6.09, 8.42) | 2.06 (1.65, 2.58) | **11A** | 9.13 (7.44, 11.22) | 3.95 (2.98, 5.23) | 11A | 4.78 (3.79, 6.03) |
| 12F | 1.46 (1.18, 1.81) | 0.41 (0.30, 0.55) | **12F** | 1.91 (1.44, 2.53) | 1.01 (0.65, 1.56) | 12F | 0.96 (0.70, 1.31) |
| 15A | 16.35 (13.45, 19.88) | 1.56 (1.19, 2.05) | 15A | 20.24 (15.92, 25.75) | 2.15 (1.52, 3.03) | 15A | 11.16 (7.91, 15.75) |
| 15C | 11.08 (9.01, 13.62) | 3.25 (2.37, 4.45) | 15C | 16.67 (12.78, 21.76) | 4.31 (2.91, 6.38) | 15C | 6.51 (4.72, 8.99) |
| 16F | 4.69 (3.75, 5.87) | 0.37 (0.28, 0.48) | 16F | 4.04 (3.19, 5.10) | 0.30 (0.22, 0.41) | 16F | 2.46 (1.78, 3.41) |
| 17F | 13.20 (11.18, 15.58) | 2.68 (2.08, 3.46) | **17F** | 14.96 (12.37, 18.08) | 7.23 (5.26, 9.92) | 17F | 9.92 (7.86, 12.53) |
| **19A** | 8.54 (7.33, 9.95) | 11.97 (9.40, 15.24) | **19A** | 9.12 (7.70, 10.81) | 12.41 (9.57, 16.11) | 19A | 8.06 (6.50, 9.98) |
| 20A | 14.83 (12.37, 17.77) | 4.93 (3.83, 6.36) | **20A** | 22.21 (17.77, 27.75) | 10.73 (7.48, 15.41) | 20A | 12.16 (8.99, 16.46) |
| **22F** | 4.41 (3.69, 5.28) | 4.33 (3.37, 5.56) | **22F** | 5.38 (4.34, 6.66) | 2.87 (2.12, 3.87) | 22F | 3.08 (2.36, 4.01) |
| 23A | 5.38 (4.19, 6.90) | 0.75 (0.55, 1.02) | 23A | 6.03 (4.55, 7.99) | 0.62 (0.43, 0.90) | 23A | 4.25 (2.88, 6.26) |
| 23B | 6.66 (5.53, 8.03) | 2.61 (1.96, 3.48) | 23B | 6.00 (4.91, 7.33) | 2.31 (1.75, 3.06) | 23B | 5.05 (3.90, 6.54) |
| 24F | 10.05 (7.59, 13.32) | 0.49 (0.39, 0.61) | 24F | 7.48 (5.45, 10.26) | 0.31 (0.23, 0.42) | 24F | 6.12 (3.84, 9.75) |
| 31 | 4.48 (3.66, 5.48) | 0.37 (0.30, 0.47) | 31 | 3.58 (2.87, 4.46) | 0.32 (0.24, 0.44) | 31 | 3.66 (2.64, 5.08) |
| **33F** | 13.36 (11.22, 15.91) | 12.72 (10.37, 15.61) | **33F** | 15.04 (12.31, 18.37) | 13.92 (10.23, 18.94) | 33F | 9.66 (7.53, 12.40) |
| 35B | 26.31 (21.60, 32.04) | 1.45 (1.18, 1.78) | 35B | 24.55 (20.09, 30.01) | 1.45 (1.17, 1.80) | 35B | 18.15 (13.61, 24.21) |

- Bold denotes pneumococcal serotypes shared between the two vaccines within a given Cohort

- Number of participants contributing to the analysis: Cohort 1 - n=175-212 for V116 group, n=91-114 for PCV15 group; Cohort 2 - n=125-161 for V116 group, n=58-76 for PPSV23 group; Cohort 3 – n=82-99

- Serotype 15C represents the immune response to the deOAc15B polysaccharide as the molecular structure for deOAc15B and 15C are similar; anti-15C immune responses are assessed in this study

- CI=confidence interval; N=number of participants randomized and vaccinated

- V116 contains all serotypes listed in Table; PCV15 contains serotypes 1, 3, 4, 5, 6A, 7F, 9V, 14, 18C, 19A, 19F, 23F, 22F, and 33F; PPSV23 contains serotypes 1, 2, 3, 4, 5, 6B, 7F, 8, 9N, 9V, 10A, 11A, 12F, 14, 15B, 17F, 18C, 19A, 19F, 20, 22F, 23F, and 33F

Supplemental Table 8: Summary of serotype-specific IgG antibody responses – Cohort 1.

| **STs** |  | **V116 (N=229)** | | **PCV15 (N=119)** | |  | **V116 (N=229)** | | **PCV15 (N=119)** | |
| --- | --- | --- | --- | --- | --- | --- | --- | --- | --- | --- |
| **Common** | Endpoint | n | Response (95% CI) | n | Response (95% CI) | **Unique** | n | Response (95% CI) | n | Response (95% CI) |
| 3 | GMC (Day 1) | 227 | 0.24 (0.21, 0.27) | 117 | 0.25 (0.21, 0.31) | 15A | 227 | 1.27 (1.07, 1.52) | 117 | 1.64 (1.24, 2.16) |
|  | GMC (Day 30) | 220 | 0.87 (0.77, 0.99) | 117 | 0.8 (0.65, 0.97) |  | 220 | 16.35 (13.45, 19.88) | 117 | 1.56 (1.19, 2.05) |
|  | GMFR | 220 | 3.6 (3.2, 4.0) | 117 | 3.1 (2.6, 3.7) |  | 220 | 12.2 (10.1, 14.7) | 117 | 1 (0.9, 1.1) |
|  | % ≥4-fold rise | 220 | 42.7% (36.1, 49.6) | 117 | 36.8% (28.0, 46.2) |  | 220 | 73.6% (67.3, 79.3) | 117 | 0.9% (0.0, 4.7) |
| 6A | GMC (Day 1) | 227 | 0.77 (0.63, 0.95) | 117 | 0.96 (0.73, 1.26) | 15C | 227 | 1.86 (1.51, 2.29) | 117 | 3.18 (2.30, 4.40) |
|  | GMC (Day 30) | 219 | 4.69 (3.72, 5.90) | 117 | 7.69 (5.60, 10.57) |  | 219 | 11.08 (9.01, 13.62) | 117 | 3.25 (2.37, 4.45) |
|  | GMFR | 219 | 5.9 (5.0, 7.1) | 117 | 8 (6.1, 10.6) |  | 219 | 5.8 (4.9, 6.9) | 117 | 1 (0.9, 1.2) |
|  | % ≥4-fold rise | 219 | 56.2% (49.3, 62.8) | 117 | 65.0% (55.6, 73.5) |  | 219 | 57.5% (50.7, 64.2) | 117 | 3.4% (0.9, 8.5) |
| 7F | GMC (Day 1) | 227 | 1.69 (1.40, 2.06) | 117 | 2.23 (1.70, 2.92) | 16F | 227 | 0.3 (0.25, 0.35) | 117 | 0.34 (0.26, 0.44) |
|  | GMC (Day 30) | 219 | 5.71 (4.89, 6.68) | 117 | 6.45 (5.08, 8.19) |  | 218 | 4.69 (3.75, 5.87) | 117 | 0.37 (0.28, 0.48) |
|  | GMFR | 219 | 3.3 (2.8, 3.8) | 117 | 2.9 (2.3, 3.5) |  | 218 | 15.2 (12.7, 18.2) | 117 | 1.1 (1.0, 1.2) |
|  | % ≥4-fold rise | 219 | 34.7% (28.4, 41.4) | 117 | 29.9% (21.8, 39.1) |  | 218 | 82.1% (76.4, 87.0) | 117 | 2.6% (0.5, 7.3) |
| 19A | GMC (Day 1) | 227 | 3.67 (3.15, 4.27) | 117 | 3.52 (2.83, 4.36) | 17F | 227 | 2.73 (2.29, 3.25) | 117 | 2.77 (2.16, 3.57) |
|  | GMC (Day 30) | 219 | 8.54 (7.33, 9.95) | 117 | 11.97 (9.40, 15.24) |  | 219 | 13.2 (11.18, 15.58) | 117 | 2.68 (2.08, 3.46) |
|  | GMFR | 219 | 2.3 (2.0, 2.7) | 117 | 3.4 (2.8, 4.2) |  | 219 | 4.7 (4.0, 5.5) | 117 | 1 (0.9, 1.1) |
|  | % ≥4-fold rise | 219 | 24.7% (19.1, 30.9) | 117 | 37.6% (28.8, 47.0) |  | 219 | 48.9% (42.1, 55.7) | 117 | 1.7% (0.2, 6.0) |
| 22F | GMC (Day 1) | 227 | 1.03 (0.86, 1.22) | 117 | 1.24 (0.94, 1.62) | 20A | 227 | 3.86 (3.21, 4.64) | 117 | 5.13 (3.97, 6.63) |
|  | GMC (Day 30) | 219 | 4.41 (3.69, 5.28) | 117 | 4.33 (3.37, 5.56) |  | 219 | 14.83 (12.37, 17.77) | 117 | 4.93 (3.83, 6.36) |
|  | GMFR | 219 | 4.2 (3.6, 4.9) | 117 | 3.4 (2.8, 4.2) |  | 219 | 3.8 (3.3, 4.4) | 117 | 1 (0.9, 1.1) |
|  | % ≥4-fold rise | 219 | 39.3% (32.8, 46.1) | 117 | 35.0% (26.5, 44.4) |  | 219 | 36.5% (30.1, 43.3) | 117 | 0.9% (0.0, 4.7) |
| 33F | GMC (Day 1) | 227 | 6.07 (5.05, 7.30) | 117 | 7.14 (5.55, 9.18) | 23A | 227 | 0.39 (0.31, 0.47) | 117 | 0.4 (0.30, 0.53) |
|  | GMC (Day 30) | 220 | 13.36 (11.22, 15.91) | 117 | 12.72 (10.37, 15.61) |  | 219 | 5.38 (4.19, 6.90) | 117 | 0.75 (0.55, 1.02) |
|  | GMFR | 220 | 2.2 (1.9, 2.5) | 117 | 1.8 (1.5, 2.1) |  | 219 | 13.5 (11.1, 16.5) | 117 | 1.8 (1.5, 2.2) |
|  | % ≥4-fold rise | 220 | 19.1% (14.1, 24.9) | 117 | 12.8% (7.4, 20.3) |  | 219 | 77.6% (71.5, 83.0) | 117 | 17.1% (10.8, 25.2) |
| **Unique** |  |  |  |  |  |  |  |  |  |  |
| 8 | GMC (Day 1) | 227 | 3.47 (2.91, 4.14) | 117 | 3.68 (2.85, 4.76) | 23B | 227 | 0.78 (0.65, 0.94) | 117 | 0.94 (0.73, 1.21) |
|  | GMC (Day 30) | 220 | 9.58 (8.21, 11.17) | 117 | 3.56 (2.74, 4.62) |  | 219 | 6.66 (5.53, 8.03) | 117 | 2.61 (1.96, 3.48) |
|  | GMFR | 220 | 2.7 (2.4, 3.1) | 117 | 1 (0.9, 1.1) |  | 219 | 8.2 (6.7, 10.0) | 117 | 2.7 (2.1, 3.5) |
|  | % ≥4-fold rise | 220 | 29.5% (23.6, 36.0) | 117 | 0.9% (0.0, 4.7) |  | 219 | 64.8% (58.1, 71.2) | 117 | 26.5% (18.8, 35.5) |
| 9N | GMC (Day 1) | 227 | 2.42 (2.02, 2.89) | 117 | 2.58 (2.01, 3.32) | 24F | 227 | 0.36 (0.30, 0.43) | 117 | 0.48 (0.38, 0.62) |
|  | GMC (Day 30) | 217 | 7.06 (5.92, 8.43) | 117 | 3.59 (2.83, 4.55) |  | 219 | 10.05 (7.59, 13.32) | 117 | 0.49 (0.39, 0.61) |
|  | GMFR | 217 | 2.9 (2.4, 3.4) | 117 | 1.4 (1.2, 1.6) |  | 219 | 26.9 (21.8, 33.1) | 117 | 1 (0.9, 1.1) |
|  | % ≥4-fold rise | 217 | 31.8% (25.7, 38.4) | 117 | 7.7% (3.6, 14.1) |  | 219 | 85.4% (80.0, 89.8) | 117 | 0.0% (0.0, 3.1) |
| 10A | GMC (Day 1) | 227 | 2.2 (1.76, 2.75) | 117 | 2.86 (2.18, 3.74) | 31 | 227 | 0.32 (0.28, 0.38) | 117 | 0.33 (0.27, 0.42) |
|  | GMC (Day 30) | 220 | 9.86 (7.99, 12.18) | 117 | 2.77 (2.12, 3.63) |  | 217 | 4.48 (3.66, 5.48) | 117 | 0.37 (0.30, 0.47) |
|  | GMFR | 220 | 4.4 (3.7, 5.2) | 117 | 1 (0.9, 1.0) |  | 217 | 13.1 (11.0, 15.6) | 117 | 1.1 (1.0, 1.2) |
|  | % ≥4-fold rise | 220 | 45.5% (38.7, 52.3) | 117 | 0.9% (0.0, 4.7) |  | 217 | 79.7% (73.8, 84.9) | 117 | 2.6% (0.5, 7.3) |
| 11A | GMC (Day 1) | 227 | 1.72 (1.49, 2.00) | 117 | 2.1 (1.68, 2.62) | 35B | 227 | 1.53 (1.33, 1.76) | 117 | 1.51 (1.24, 1.84) |
|  | GMC (Day 30) | 219 | 7.16 (6.09, 8.42) | 117 | 2.06 (1.65, 2.58) |  | 218 | 26.31 (21.60, 32.04) | 117 | 1.45 (1.18, 1.78) |
|  | GMFR | 219 | 4.1 (3.6, 4.7) | 117 | 1 (0.9, 1.0) |  | 218 | 16.8 (13.9, 20.3) | 117 | 1 (0.9, 1.0) |
|  | % ≥4-fold rise | 219 | 43.8% (37.2, 50.7) | 117 | 0.0% (0.0, 3.1) |  | 218 | 83.9% (78.4, 88.6) | 117 | 0.0% (0.0, 3.1) |
| 12F | GMC (Day 1) | 227 | 0.32 (0.26, 0.39) | 117 | 0.4 (0.29, 0.54) |  |  |  |  |  |
|  | GMC (Day 30) | 220 | 1.46 (1.18, 1.81) | 117 | 0.41 (0.30, 0.55) |  |  |  |  |  |
|  | GMFR | 220 | 4.2 (3.6, 5.0) | 117 | 1 (0.9, 1.1) |  |  |  |  |  |
|  | % ≥4-fold rise | 220 | 44.5% (37.9, 51.4) | 117 | 2.6% (0.5, 7.3) |  |  |  |  |  |

Supplemental Table 9: Summary of serotype-specific IgG antibody responses – Cohort 2.

| **STs** |  | **V116 (N=174)** | | **PPSV23 (N=85)** | |  | **V116 (N=174)** | | **PPSV23 (N=85)** | |
| --- | --- | --- | --- | --- | --- | --- | --- | --- | --- | --- |
| **Common** | Endpoint | n | Response (95% CI) | n | Response (95% CI) | **Unique** | n | Response (95% CI) | n | Response (95% CI) |
| 3 | GMC (Day 1) | 171 | 0.23 (0.20, 0.27) | 84 | 0.27 (0.20, 0.35) | 6A | 171 | 2.39 (1.85, 3.08) | 84 | 2.05 (1.38, 3.06) |
|  | GMC (Day 30) | 167 | 0.89 (0.77, 1.03) | 81 | 1.58 (1.25, 2.00) |  | 167 | 7.81 (6.30, 9.68) | 81 | 5.24 (3.62, 7.56) |
|  | GMFR | 167 | 3.7 (3.2, 4.3) | 81 | 5.7 (4.3, 7.4) |  | 167 | 3.3 (2.7, 4.0) | 81 | 2.5 (1.9, 3.2) |
|  | % ≥4-fold rise | 167 | 40.7% (33.2, 48.6) | 81 | 54.3% (42.9, 65.4) |  | 167 | 35.9% (28.7, 43.7) | 81 | 27.2% (17.9, 38.2) |
| 7F | GMC (Day 1) | 171 | 1.74 (1.40, 2.18) | 84 | 2.6 (2.00, 3.39) | 15A | 171 | 0.81 (0.65, 1.00) | 84 | 0.85 (0.63, 1.16) |
|  | GMC (Day 30) | 167 | 5.65 (4.75, 6.73) | 81 | 8.34 (6.57, 10.59) |  | 167 | 20.24 (15.92, 25.75) | 81 | 2.15 (1.52, 3.03) |
|  | GMFR | 167 | 3.2 (2.7, 3.8) | 81 | 3.2 (2.6, 3.9) |  | 167 | 24.5 (19.7, 30.3) | 81 | 2.4 (2.0, 3.0) |
|  | % ≥4-fold rise | 167 | 37.7% (30.4, 45.5) | 81 | 33.3% (23.2, 44.7) |  | 167 | 89.8% (84.2, 94.0) | 81 | 28.4% (18.9, 39.5) |
| 8 | GMC (Day 1) | 171 | 0.92 (0.74, 1.15) | 84 | 0.95 (0.69, 1.30) | 15C | 171 | 0.87 (0.68, 1.12) | 84 | 0.68 (0.50, 0.94) |
|  | GMC (Day 30) | 167 | 11.29 (9.28, 13.73) | 81 | 14.47 (10.86, 19.27) |  | 166 | 16.67 (12.78, 21.76) | 81 | 4.31 (2.91, 6.38) |
|  | GMFR | 167 | 12.1 (9.8, 15.0) | 81 | 14.4 (10.3, 20.0) |  | 166 | 18.8 (14.9, 23.8) | 81 | 6.2 (4.8, 8.2) |
|  | % ≥4-fold rise | 167 | 78.4% (71.4, 84.4) | 81 | 76.5% (65.8, 85.2) |  | 166 | 80.1% (73.2, 85.9) | 81 | 64.2% (52.8, 74.6) |
| 9N | GMC (Day 1) | 171 | 0.97 (0.79, 1.20) | 84 | 0.78 (0.59, 1.04) | 16F | 171 | 0.24 (0.20, 0.29) | 84 | 0.19 (0.15, 0.25) |
|  | GMC (Day 30) | 167 | 8.48 (6.89, 10.44) | 81 | 7.32 (5.47, 9.78) |  | 166 | 4.04 (3.19, 5.10) | 81 | 0.3 (0.22, 0.41) |
|  | GMFR | 167 | 8.7 (7.1, 10.6) | 81 | 9.1 (6.8, 12.1) |  | 166 | 15.7 (12.9, 19.0) | 81 | 1.6 (1.3, 1.8) |
|  | % ≥4-fold rise | 167 | 68.9% (61.2, 75.8) | 81 | 72.8% (61.8, 82.1) |  | 166 | 84.9% (78.6, 90.0) | 81 | 13.6% (7.0, 23.0) |
| 10A | GMC (Day 1) | 171 | 0.93 (0.73, 1.19) | 84 | 0.72 (0.52, 0.98) | 23A | 171 | 0.31 (0.25, 0.38) | 84 | 0.26 (0.19, 0.35) |
|  | GMC (Day 30) | 167 | 18.21 (14.30, 23.18) | 81 | 7.29 (4.94, 10.74) |  | 166 | 6.03 (4.55, 7.99) | 81 | 0.62 (0.43, 0.90) |
|  | GMFR | 167 | 18.9 (15.3, 23.4) | 81 | 10.1 (7.6, 13.4) |  | 166 | 18.9 (15.3, 23.4) | 81 | 2.3 (1.9, 2.7) |
|  | % ≥4-fold rise | 167 | 82.6% (76.0, 88.1) | 81 | 80.2% (69.9, 88.3) |  | 166 | 86.7% (80.6, 91.5) | 81 | 21.0% (12.7, 31.5) |
| 11A | GMC (Day 1) | 171 | 0.87 (0.73, 1.04) | 84 | 0.68 (0.52, 0.88) | 23B | 171 | 0.81 (0.66, 0.99) | 84 | 0.98 (0.73, 1.33) |
|  | GMC (Day 30) | 167 | 9.13 (7.44, 11.22) | 81 | 3.95 (2.98, 5.23) |  | 167 | 6 (4.91, 7.33) | 81 | 2.31 (1.75, 3.06) |
|  | GMFR | 167 | 10.3 (8.5, 12.6) | 81 | 5.6 (4.4, 7.2) |  | 167 | 7.3 (6.0, 8.9) | 81 | 2.3 (1.9, 2.9) |
|  | % ≥4-fold rise | 167 | 72.5% (65.0, 79.1) | 81 | 63.0% (51.5, 73.4) |  | 167 | 60.5% (52.6, 67.9) | 81 | 21.0% (12.7, 31.5) |
| 12F | GMC (Day 1) | 171 | 0.11 (0.09, 0.14) | 84 | 0.1 (0.08, 0.14) | 24F | 171 | 0.34 (0.28, 0.41) | 84 | 0.3 (0.21, 0.41) |
|  | GMC (Day 30) | 167 | 1.91 (1.44, 2.53) | 81 | 1.01 (0.65, 1.56) |  | 167 | 7.48 (5.45, 10.26) | 81 | 0.31 (0.23, 0.42) |
|  | GMFR | 167 | 14.2 (11.3, 17.9) | 81 | 8.4 (5.8, 12.0) |  | 167 | 21.7 (17.3, 27.2) | 81 | 1 (0.9, 1.1) |
|  | % ≥4-fold rise | 167 | 75.4% (68.2, 81.8) | 81 | 60.5% (49.0, 71.2) |  | 167 | 88.0% (82.1, 92.5) | 81 | 0.0% (0.0, 4.5) |
| 17F | GMC (Day 1) | 171 | 0.87 (0.72, 1.06) | 84 | 0.69 (0.52, 0.90) | 31 | 171 | 0.22 (0.18, 0.27) | 84 | 0.21 (0.16, 0.26) |
|  | GMC (Day 30) | 167 | 14.96 (12.37, 18.08) | 81 | 7.23 (5.26, 9.92) |  | 167 | 3.58 (2.87, 4.46) | 81 | 0.32 (0.24, 0.44) |
|  | GMFR | 167 | 17.4 (14.3, 21.1) | 81 | 10.5 (7.8, 14.1) |  | 167 | 14.4 (11.9, 17.3) | 81 | 1.5 (1.3, 1.8) |
|  | % ≥4-fold rise | 167 | 83.8% (77.4, 89.1) | 81 | 74.1% (63.1, 83.2) |  | 167 | 84.4% (78.0, 89.6) | 81 | 11.1% (5.2, 20.0) |
| 19A | GMC (Day 1) | 171 | 4.12 (3.40, 5.01) | 84 | 4.22 (3.20, 5.56) | 35B | 171 | 1.52 (1.27, 1.82) | 84 | 1.52 (1.23, 1.88) |
|  | GMC (Day 30) | 167 | 9.12 (7.70, 10.81) | 81 | 12.41 (9.57, 16.11) |  | 167 | 24.55 (20.09, 30.01) | 81 | 1.45 (1.17, 1.80) |
|  | GMFR | 167 | 2.2 (1.9, 2.5) | 81 | 3 (2.4, 3.6) |  | 167 | 15.6 (12.9, 18.7) | 81 | 0.9 (0.9, 1.0) |
|  | % ≥4-fold rise | 167 | 19.8% (14.0, 26.6) | 81 | 33.3% (23.2, 44.7) |  | 167 | 82.6% (76.0, 88.1) | 81 | 0.0% (0.0, 4.5) |
| 20A | GMC (Day 1) | 171 | 1.52 (1.28, 1.82) | 84 | 1.21 (0.93, 1.58) |  |  |  |  |  |
|  | GMC (Day 30) | 167 | 22.21 (17.77, 27.75) | 81 | 10.73 (7.48, 15.41) |  |  |  |  |  |
|  | GMFR | 167 | 14.8 (12.2, 17.9) | 81 | 8.5 (6.4, 11.4) |  |  |  |  |  |
|  | % ≥4-fold rise | 167 | 83.2% (76.7, 88.6) | 81 | 69.1% (57.9, 78.9) |  |  |  |  |  |
| 22F | GMC (Day 1) | 171 | 0.33 (0.27, 0.41) | 84 | 0.29 (0.21, 0.40) |  |  |  |  |  |
|  | GMC (Day 30) | 167 | 5.38 (4.34, 6.66) | 81 | 2.87 (2.12, 3.87) |  |  |  |  |  |
|  | GMFR | 167 | 14.3 (11.4, 18.0) | 81 | 8.5 (6.2, 11.6) |  |  |  |  |  |
|  | % ≥4-fold rise | 167 | 78.4% (71.4, 84.4) | 81 | 65.4% (54.0, 75.7) |  |  |  |  |  |
| 33F | GMC (Day 1) | 171 | 1.7 (1.38, 2.10) | 84 | 1.32 (0.98, 1.78) |  |  |  |  |  |
|  | GMC (Day 30) | 167 | 15.04 (12.31, 18.37) | 81 | 13.92 (10.23, 18.94) |  |  |  |  |  |
|  | GMFR | 167 | 8.6 (7.1, 10.5) | 81 | 10.5 (7.9, 13.9) |  |  |  |  |  |
|  | % ≥4-fold rise | 167 | 72.5% (65.0, 79.1) | 81 | 75.3% (64.5, 84.2) |  |  |  |  |  |

Supplemental Table 10: Summary of serotype-specific IgG antibody responses – Cohort 3.

|  |  | **V116 (N=105)** | |  | **V116 (N=105)** | |
| --- | --- | --- | --- | --- | --- | --- |
| **Serotype** | Endpoint | n | Response (95% CI) | Serotype | n | Response (95% CI) |
| 3 | GMC (Day 1) | 105 | 0.29 (0.23, 0.36) | 17F | 105 | 2.57 (1.97, 3.36) |
|  | GMC (Day 30) | 99 | 0.85 (0.70, 1.03) |  | 99 | 9.92 (7.86, 12.53) |
|  | GMFR | 99 | 2.9 (2.3, 3.5) |  | 99 | 3.8 (2.9, 5.0) |
|  | % ≥4-fold rise | 99 | 35.4% (26.0, 45.6) |  | 99 | 44.4% (34.5, 54.8) |
| 6A | GMC (Day 1) | 105 | 1.19 (0.90, 1.58) | 19A | 105 | 3.93 (3.07, 5.02) |
|  | GMC (Day 30) | 99 | 5.48 (4.27, 7.03) |  | 99 | 8.06 (6.50, 9.98) |
|  | GMFR | 99 | 4.4 (3.4, 5.7) |  | 99 | 2 (1.7, 2.4) |
|  | % ≥4-fold rise | 99 | 45.5% (35.4, 55.8) |  | 99 | 19.2% (12.0, 28.3) |
| 7F | GMC (Day 1) | 105 | 1.5 (1.12, 2.02) | 20A | 105 | 3.31 (2.45, 4.47) |
|  | GMC (Day 30) | 99 | 5.07 (4.12, 6.23) |  | 99 | 12.16 (8.99, 16.46) |
|  | GMFR | 99 | 3.2 (2.6, 4.0) |  | 99 | 3.7 (2.9, 4.8) |
|  | % ≥4-fold rise | 99 | 37.4% (27.9, 47.7) |  | 99 | 49.5% (39.3, 59.7) |
| 8 | GMC (Day 1) | 105 | 3.17 (2.38, 4.22) | 22F | 105 | 0.84 (0.60, 1.18) |
|  | GMC (Day 30) | 99 | 7.48 (5.87, 9.53) |  | 99 | 3.08 (2.36, 4.01) |
|  | GMFR | 99 | 2.3 (1.9, 2.8) |  | 99 | 3.5 (2.7, 4.6) |
|  | % ≥4-fold rise | 99 | 26.3% (17.9, 36.1) |  | 99 | 43.4% (33.5, 53.8) |
| 9N | GMC (Day 1) | 105 | 1.31 (1.00, 1.72) | 23A | 105 | 0.32 (0.23, 0.44) |
|  | GMC (Day 30) | 99 | 4.15 (3.23, 5.34) |  | 99 | 4.25 (2.88, 6.26) |
|  | GMFR | 99 | 3.2 (2.6, 3.9) |  | 99 | 13.1 (9.6, 17.7) |
|  | % ≥4-fold rise | 99 | 36.4% (26.9, 46.6) |  | 99 | 77.8% (68.3, 85.5) |
| 10A | GMC (Day 1) | 105 | 2.36 (1.66, 3.36) | 23B | 105 | 0.67 (0.51, 0.88) |
|  | GMC (Day 30) | 99 | 10.03 (7.21, 13.98) |  | 99 | 5.05 (3.90, 6.54) |
|  | GMFR | 99 | 4.2 (3.1, 5.5) |  | 99 | 7.3 (5.4, 9.7) |
|  | % ≥4-fold rise | 99 | 46.5% (36.4, 56.8) |  | 99 | 64.6% (54.4, 74.0) |
| 11A | GMC (Day 1) | 105 | 1.35 (1.06, 1.72) | 24F | 105 | 0.24 (0.18, 0.32) |
|  | GMC (Day 30) | 99 | 4.78 (3.79, 6.03) |  | 99 | 6.12 (3.84, 9.75) |
|  | GMFR | 99 | 3.5 (2.7, 4.3) |  | 99 | 24.4 (17.1, 34.9) |
|  | % ≥4-fold rise | 99 | 43.4% (33.5, 53.8) |  | 99 | 83.8% (75.1, 90.5) |
| 12F | GMC (Day 1) | 105 | 0.25 (0.18, 0.35) | 31 | 105 | 0.26 (0.19, 0.34) |
|  | GMC (Day 30) | 99 | 0.96 (0.70, 1.31) |  | 99 | 3.66 (2.64, 5.08) |
|  | GMFR | 99 | 3.5 (2.7, 4.5) |  | 99 | 13 (9.9, 17.0) |
|  | % ≥4-fold rise | 99 | 39.4% (29.7, 49.7) |  | 99 | 80.8% (71.7, 88.0) |
| 15A | GMC (Day 1) | 105 | 0.66 (0.49, 0.90) | 33F | 105 | 3.95 (2.94, 5.32) |
|  | GMC (Day 30) | 99 | 11.16 (7.91, 15.75) |  | 99 | 9.66 (7.53, 12.40) |
|  | GMFR | 99 | 16.6 (12.0, 22.9) |  | 99 | 2.4 (1.9, 3.0) |
|  | % ≥4-fold rise | 99 | 77.8% (68.3, 85.5) |  | 99 | 27.3% (18.8, 37.1) |
| 15C | GMC (Day 1) | 105 | 1.01 (0.73, 1.40) | 35B | 105 | 1.15 (0.91, 1.46) |
|  | GMC (Day 30) | 99 | 6.51 (4.72, 8.99) |  | 99 | 18.15 (13.61, 24.21) |
|  | GMFR | 99 | 6.4 (5.0, 8.1) |  | 99 | 15.7 (11.9, 20.8) |
|  | % ≥4-fold rise | 99 | 66.7% (56.5, 75.8) |  | 99 | 84.8% (76.2, 91.3) |
| 16F | GMC (Day 1) | 105 | 0.19 (0.15, 0.24) |  |  |  |
|  | GMC (Day 30) | 99 | 2.46 (1.78, 3.41) |  |  |  |
|  | GMFR | 99 | 11.9 (9.2, 15.4) |  |  |  |
|  | % ≥4-fold rise | 99 | 77.8% (68.3, 85.5) |  |  |  |

Supplemental Table 11: Serotype-specific OPA GMTs by age - Cohort 1.

|  | **V116** | | | | **PCV15** | | | |
| --- | --- | --- | --- | --- | --- | --- | --- | --- |
|  | 50-64 years (N=48) | | ≥65 years (N=181) | | 50-64 years (N=25) | | ≥65 years (N=94) | |
| Common STs | n | Observed GMT (95% CI) | n | Observed GMT (95% CI) | n | Observed GMT (95% CI) | n | Observed GMT (95% CI) |
| 3 | 38 | 377.4 (269.2, 528.9) | 159 | 240.3 (201.4, 286.6) | 21 | 278.3 (168.6, 459.1) | 82 | 214.7 (167.9, 274.5) |
| 6A | 39 | 2320.3 (1485.7, 3624.0) | 152 | 1515.8 (1203.1, 1909.8) | 20 | 3171.1 (1665.1, 6039.4) | 74 | 1851.5 (1356.9, 2526.5) |
| 7F | 42 | 2830.1 (2125.4, 3768.5) | 167 | 2046.7 (1735.2, 2414.1) | 23 | 1762.3 (987.3, 3145.5) | 87 | 1747.2 (1375.7, 2219.0) |
| 19A | 40 | 1713.7 (1288.2, 2279.7) | 164 | 1468.6 (1253.7, 1720.4) | 23 | 2787.3 (1637.9, 4743.4) | 86 | 1856.7 (1471.0, 2343.5) |
| 22F | 42 | 3346.1 (2344.7, 4775.1) | 164 | 1735.2 (1466.2, 2053.6) | 20 | 1939.4 (1123.1, 3348.9) | 88 | 1526.3 (1129.3, 2062.9) |
| 33F | 39 | 6946.1 (4638.7, 10401.5) | 149 | 3806.0 (3150.4, 4598.0) | 20 | 4578.7 (2339.8, 8960.0) | 79 | 3149.9 (2432.9, 4078.2) |
| Unique STs |  |  |  |  |  |  |  |  |
| 8 | 44 | 1558.5 (1173.7, 2069.5) | 164 | 1205.8 (1037.6, 1401.2) | 23 | 392.3 (158.4, 971.5) | 90 | 334.9 (237.7, 471.8) |
| 9N | 37 | 4694.4 (3548.4, 6210.4) | 154 | 3617.9 (3101.7, 4220.1) | 24 | 2527.2 (1651.7, 3866.8) | 87 | 2088.6 (1696.3, 2571.7) |
| 10A | 44 | 2819.9 (1832.6, 4339.1) | 165 | 1809.0 (1457.9, 2244.7) | 24 | 739.8 (347.9, 1573.1) | 88 | 412.5 (286.1, 594.7) |
| 11A | 36 | 2682.4 (1993.0, 3610.3) | 161 | 1871.3 (1549.6, 2259.7) | 22 | 1086.8 (623.2, 1895.4) | 78 | 240.9 (154.9, 374.7) |
| 12F | 44 | 2492.4 (1748.1, 3553.7) | 168 | 769.3 (593.1, 997.7) | 24 | 53.7 (20.6, 140.1) | 90 | 89.7 (57.5, 139.9) |
| 15A | 33 | 5621.8 (4039.2, 7824.4) | 142 | 3907.4 (3237.1, 4716.6) | 19 | 684.7 (341.9, 1371.3) | 74 | 934.8 (618.7, 1412.5) |
| 15C | 42 | 4136.9 (3053.9, 5603.9) | 164 | 1987.4 (1559.3, 2533.2) | 23 | 415.4 (172.0, 1003.3) | 87 | 578.3 (379.9, 880.2) |
| 16F | 38 | 4243.7 (2970.9, 6061.6) | 149 | 2815.8 (2388.6, 3319.3) | 22 | 854.9 (461.6, 1583.4) | 85 | 320.7 (241.9, 425.2) |
| 17F | 38 | 5437.9 (4380.2, 6750.9) | 156 | 3255.7 (2771.9, 3823.9) | 22 | 1336.4 (937.9, 1904.3) | 86 | 858.6 (592.8, 1243.7) |
| 20A | 38 | 3650.8 (2467.5, 5401.6) | 157 | 2681.1 (2258.5, 3182.7) | 24 | 1758.8 (1101.5, 2808.5) | 86 | 919.1 (694.6, 1216.2) |
| 23A | 41 | 3048.2 (1902.0, 4885.2) | 161 | 2215.7 (1676.1, 2929.1) | 18 | 653.6 (232.1, 1840.5) | 73 | 258.1 (161.0, 413.7) |
| 23B | 38 | 873.6 (511.5, 1492.1) | 159 | 632.6 (467.5, 856.0) | 22 | 173.4 (62.3, 482.7) | 88 | 148.2 (90.4, 243.0) |
| 24F | 42 | 2529.5 (1400.5, 4568.8) | 159 | 1671.5 (1257.4, 2221.8) | 20 | 228.9 (72.4, 723.7) | 77 | 87.4 (55.8, 136.8) |
| 31 | 36 | 4698.5 (2808.6, 7860.2) | 158 | 2728.9 (2202.7, 3380.9) | 22 | 106.4 (36.5, 310.3) | 86 | 115.0 (72.5, 182.4) |
| 35B | 36 | 10038.3 (7228.7, 13939.8) | 158 | 6113.9 (5131.1, 7284.8) | 22 | 1608.2 (874.3, 2958.4) | 85 | 905.6 (624.1, 1314.2) |

Supplemental Table 12: Serotype-specific OPA GMTs by age - Cohort 2.

|  | **V116** | | | | **PPSV23** | | | |
| --- | --- | --- | --- | --- | --- | --- | --- | --- |
|  | 50-64 years (N=80) | | ≥65 years (N=94) | | 50-64 years (N=39) | | ≥65 years (N=46) | |
| Common STs | n | Observed GMT (95% CI) | n | Observed GMT (95% CI) | n | Observed GMT (95% CI) | n | Observed GMT (95% CI) |
| 3 | 66 | 423.7 (337.8, 531.4) | 83 | 367 (291.6, 462.0) | 35 | 562 (412.1, 766.4) | 40 | 602.2 (404.4, 896.6) |
| 7F | 69 | 3018.7 (2295.6, 3969.8) | 81 | 3227.7 (2519.6, 4134.7) | 34 | 3517 (2587.4, 4780.5) | 36 | 4643 (3241.9, 6649.7) |
| 8 | 73 | 2383 (1835.0, 3094.6) | 88 | 2269.2 (1881.8, 2736.4) | 36 | 3013.7 (2241.2, 4052.5) | 39 | 2480 (1802.6, 3411.8) |
| 9N | 64 | 6198.8 (4826.4, 7961.4) | 79 | 8157.9 (6402.9, 10394.0) | 29 | 5939.4 (4134.3, 8532.6) | 29 | 7075.4 (4545.1, 11014.4) |
| 10A | 72 | 4365.3 (3379.5, 5638.8) | 83 | 3667.8 (2925.7, 4598.2) | 37 | 2081.3 (1296.1, 3342.2) | 36 | 1546.3 (683.1, 3500.2) |
| 11A | 59 | 2911.6 (2183.8, 3881.9) | 83 | 2801.3 (2287.6, 3430.3) | 34 | 1641.5 (1227.7, 2194.9) | 37 | 1828.9 (1242.4, 2692.3) |
| 12F | 75 | 2772.2 (2075.6, 3702.7) | 85 | 2373.4 (1861.9, 3025.3) | 37 | 1532.1 (856.7, 2740.0) | 36 | 1280.7 (659.4, 2487.4) |
| 17F | 53 | 6673.8 (5169.6, 8615.8) | 72 | 5489.9 (4368.3, 6899.6) | 29 | 4580.5 (3280.5, 6395.7) | 38 | 4211.3 (2847.5, 6228.5) |
| 19A | 73 | 2521.8 (2036.8, 3122.4) | 85 | 2535.1 (2107.0, 3050.1) | 35 | 3531.2 (2711.7, 4598.3) | 39 | 3001.9 (2194.8, 4105.6) |
| 20A | 59 | 5630.1 (4153.0, 7632.4) | 79 | 6302.1 (4814.2, 8249.9) | 36 | 4272.2 (2940.4, 6207.1) | 36 | 2696.2 (1715.9, 4236.7) |
| 22F | 63 | 5040.7 (3675.3, 6913.2) | 80 | 3935.9 (2927.3, 5292.1) | 34 | 2435.5 (1605.5, 3694.6) | 37 | 2608.2 (1586.5, 4287.7) |
| 33F | 62 | 9582.9 (6792.4, 13519.9) | 69 | 7067.4 (5006.1, 9977.4) | 28 | 6606.3 (4293.1, 10166.0) | 31 | 11307.6 (6491.8, 19695.9) |
| Unique STs |  |  |  |  |  |  |  |  |
| 6A | 68 | 3593.5 (2829.2, 4564.3) | 84 | 3648.9 (2954.7, 4506.2) | 36 | 2950.8 (2027.2, 4295.2) | 38 | 1141.9 (592.4, 2201.1) |
| 15A | 63 | 5828.5 (4418.7, 7688.3) | 71 | 6520 (5162.2, 8235.0) | 31 | 1597.2 (1057.0, 2413.4) | 32 | 1740 (1097.9, 2757.5) |
| 15C | 71 | 4330.8 (3252.7, 5766.2) | 81 | 4337.6 (3297.9, 5705.0) | 34 | 1739 (945.1, 3199.8) | 38 | 1265.4 (717.0, 2233.3) |
| 16F | 66 | 5226.9 (4077.7, 6700.0) | 80 | 4183.4 (3222.0, 5431.8) | 36 | 691.1 (409.4, 1166.8) | 38 | 993.6 (669.2, 1475.4) |
| 23A | 71 | 4141.6 (2997.2, 5723.1) | 85 | 4349 (3212.1, 5888.3) | 28 | 615.9 (282.2, 1344.4) | 32 | 318.9 (139.8, 727.2) |
| 23B | 73 | 1253.8 (864.9, 1817.6) | 87 | 1809.7 (1299.1, 2521.1) | 37 | 321.3 (174.3, 592.1) | 38 | 130.9 (64.7, 265.1) |
| 24F | 69 | 2378.5 (1722.6, 3284.3) | 82 | 3099.1 (2436.8, 3941.4) | 27 | 66.9 (28.0, 159.7) | 36 | 38 (19.3, 75.0) |
| 31 | 66 | 4533.3 (3255.8, 6312.1) | 80 | 4317 (3169.5, 5879.9) | 33 | 221.3 (100.5, 487.3) | 35 | 135.4 (62.3, 294.4) |
| 35B | 67 | 8950.6 (6783.6, 11809.8) | 81 | 7531.2 (5836.2, 9718.4) | 36 | 1301.8 (863.2, 1963.1) | 40 | 1764.3 (1232.0, 2526.5) |

Supplemental Table 13: Serotype-specific OPA GMTs by age - Cohort 3.

|  | **V116** | | | |
| --- | --- | --- | --- | --- |
|  | 50-64 years (N=17) | | ≥65 years (N=88) | |
| Serotype | n | Observed GMT (95% CI) | n | Observed GMT (95% CI) |
| 3 | 15 | 173.7 (102.3, 295.0) | 70 | 362.4 (277.7, 472.9) |
| 6A | 15 | 2240.6 (1181.2, 4250.2) | 78 | 2070.8 (1644.5, 2607.6) |
| 7F | 17 | 1944.6 (1261.7, 2997.0) | 79 | 2075 (1589.0, 2709.7) |
| 8 | 17 | 1491.3 (834.4, 2665.6) | 81 | 1485.9 (1215.5, 1816.5) |
| 9N | 14 | 4009.2 (2392.4, 6718.7) | 76 | 4062.9 (3342.3, 4938.8) |
| 10A | 16 | 1746 (591.6, 5153.4) | 80 | 2768.8 (2148.0, 3569.0) |
| 11A | 15 | 2455 (1232.9, 4888.4) | 72 | 2356.3 (1867.4, 2973.1) |
| 12F | 17 | 1315.7 (651.3, 2657.8) | 82 | 1219.3 (911.3, 1631.4) |
| 15A | 11 | 3697.8 (1932.0, 7077.4) | 75 | 4429.8 (3372.9, 5817.8) |
| 15C | 13 | 1943.9 (1143.8, 3303.7) | 76 | 2237.4 (1528.3, 3275.4) |
| 16F | 14 | 1930.6 (639.9, 5824.5) | 75 | 2595 (1991.1, 3382.1) |
| 17F | 14 | 2929.9 (1636.1, 5246.9) | 68 | 4055.7 (3163.8, 5198.9) |
| 19A | 15 | 1188.5 (739.5, 1910.2) | 78 | 1610.9 (1311.0, 1979.4) |
| 20A | 16 | 1170.9 (562.2, 2438.3) | 72 | 2863 (2197.9, 3729.4) |
| 22F | 17 | 1891 (1084.5, 3297.2) | 82 | 1918.1 (1398.4, 2631.0) |
| 23A | 15 | 3121.5 (986.9, 9872.9) | 71 | 4173.4 (2856.6, 6097.1) |
| 23B | 17 | 706 (301.7, 1652.1) | 80 | 876.7 (610.2, 1259.6) |
| 24F | 15 | 1087.4 (356.2, 3319.5) | 75 | 2315.6 (1710.8, 3134.3) |
| 31 | 16 | 2153.5 (1038.7, 4465.0) | 74 | 3599.7 (2653.0, 4884.1) |
| 33F | 15 | 5077.4 (2774.6, 9291.5) | 73 | 4571.8 (3338.5, 6260.7) |
| 35B | 17 | 5555.6 (3380.6, 9130.0) | 73 | 5904.3 (4606.9, 7567.1) |

Supplemental Figure 2: Serotype-specific OPA GMTs by time since last pneumococcal vaccination – Cohort 1.


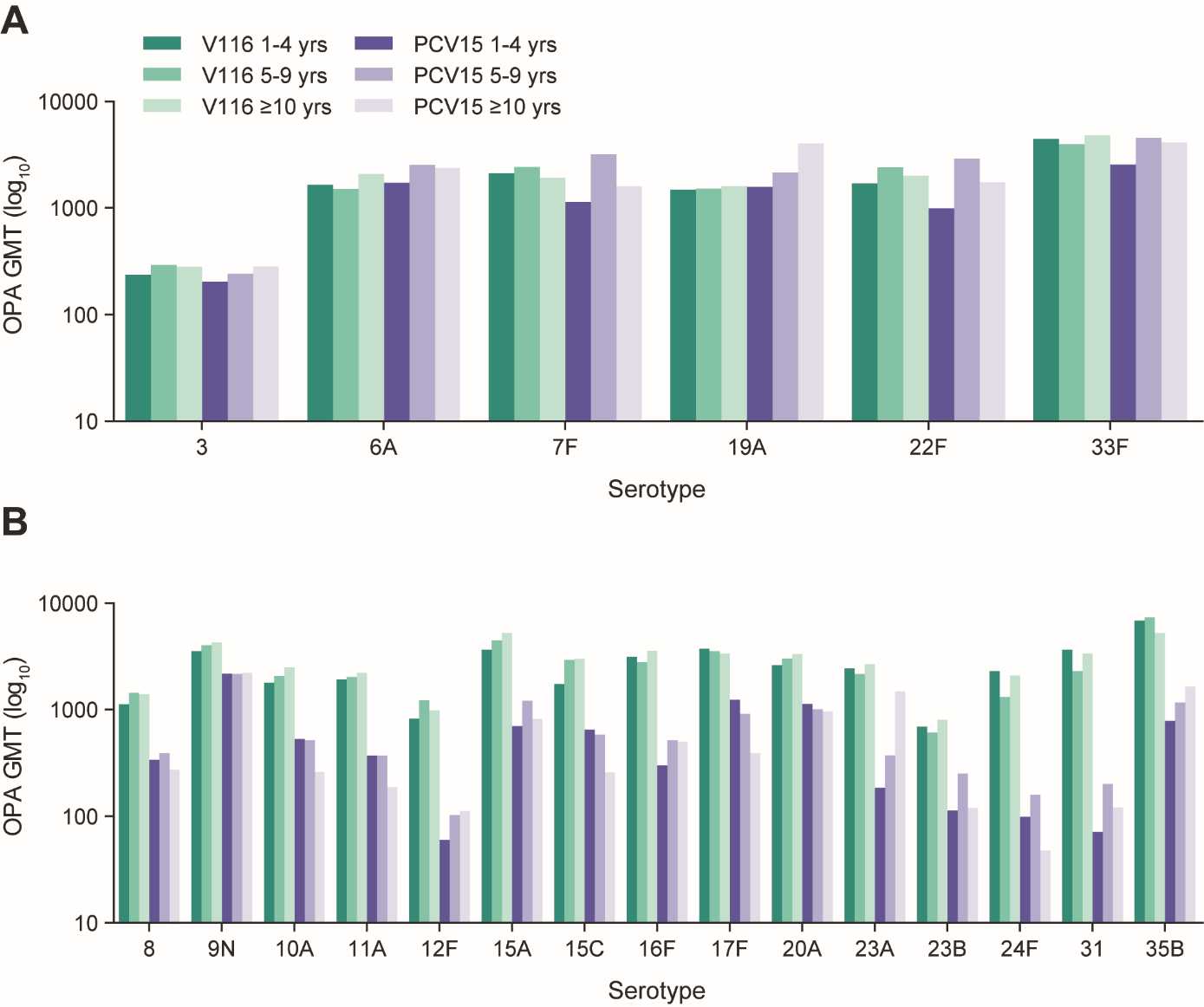


Supplemental Figure 3: Serotype-specific OPA GMTs by time since last pneumococcal vaccination – Cohort 2.


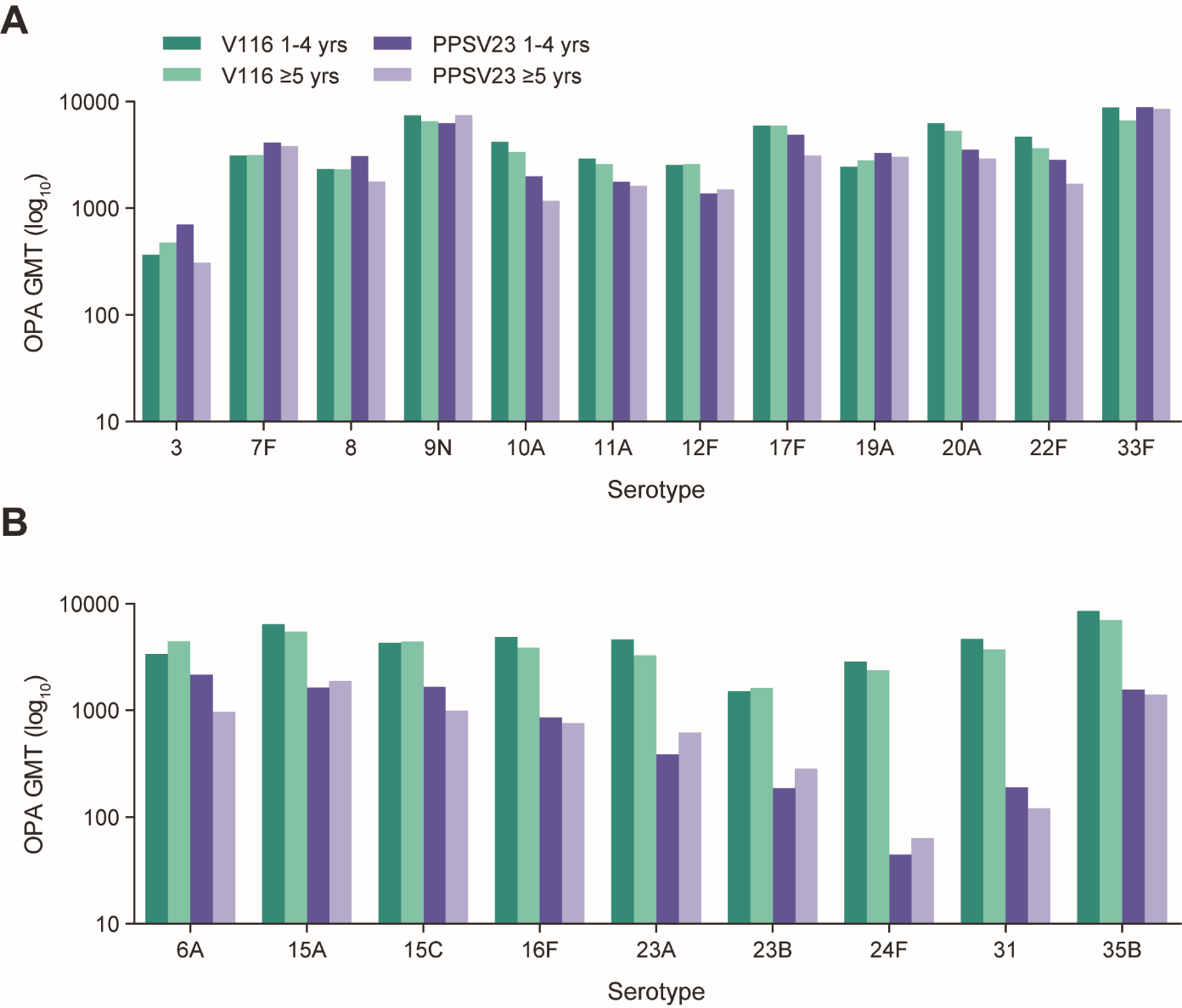


Supplemental Figure 4: Serotype-specific OPA GMTs by time since last pneumococcal vaccination – Cohort 3.


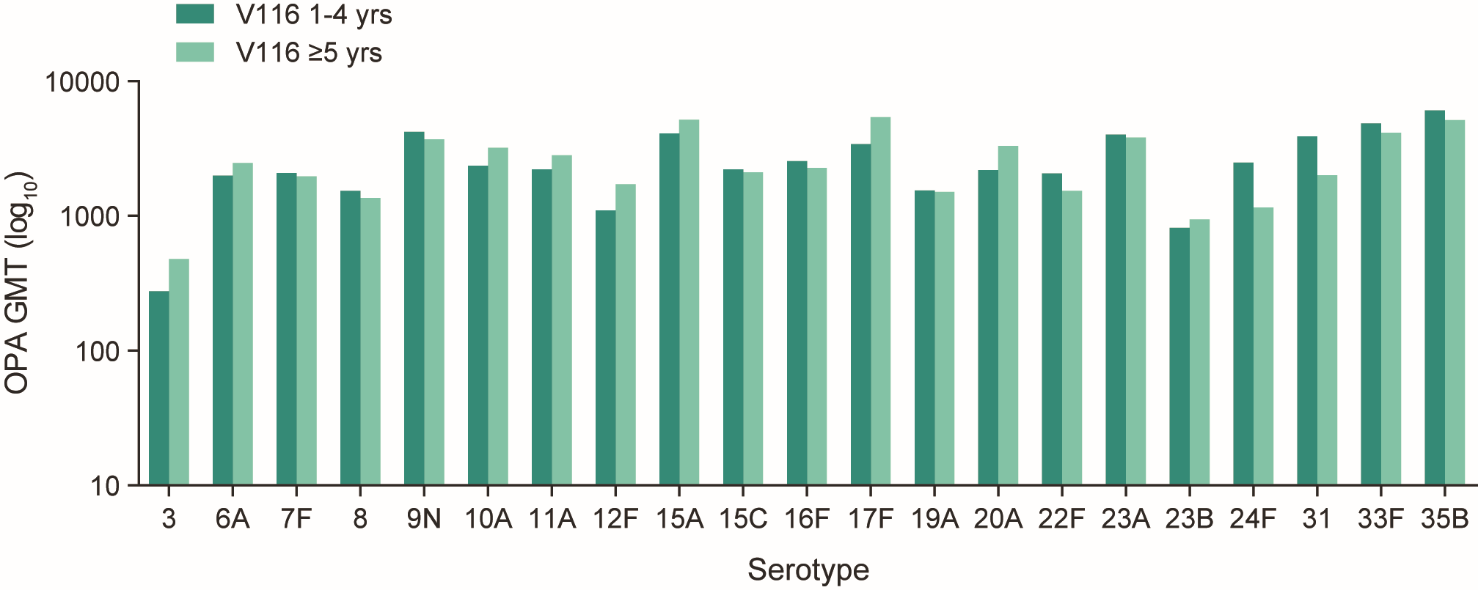


Supplemental Table 14: Solicited AEs by duration.

|  | **Cohort 1** | | **Cohort 2** | | **Cohort 3** |
| --- | --- | --- | --- | --- | --- |
|  | **V116 (n=230)** | **PCV15 (n=117)** | **V116 (n=174)** | **PPSV23 (n=85)** | **V116 (n=105)** |
|  | n (%) | n (%) | n (%) | n (%) | n (%) |
| **Solicited injection-site AEs** |  |  |  |  |  |
| Erythema | 17 (7.4) | 9 (7.7) | 13 (7.5) | 8 (9.4) | 8 (7.6) |
| ≤3 days | 15 (6.5) | 7 (6.0) | 12 (6.9) | 8 (9.4) | 5 (4.8) |
| >3 days and ≤5 days | 2 (0.9) | 1 (0.9) | 0 (0.0) | 0 (0.0) | 3 (2.9) |
| >5 days and ≤10 days | 0 (0.0) | 1 (0.9) | 1 (0.6) | 0 (0.0) | 0 (0.0) |
| Pain | 82 (35.7) | 51 (43.6) | 72 (41.4) | 40 (47.1) | 46 (43.8) |
| ≤3 days | 77 (33.5) | 45 (38.5) | 64 (36.8) | 34 (40.0) | 39 (37.1) |
| >3 days and ≤5 days | 4 (1.7) | 5 (4.3) | 8 (4.6) | 6 (7.1) | 7 (6.7) |
| >5 days and ≤10 days | 1 (0.4) | 0 (0.0) | 0 (0.0) | 0 (0.0) | 0 (0.0) |
| >10 days | 0 (0.0) | 1 (0.9) | 0 (0.0) | 0 (0.0) | 0 (0.0) |
| Swelling | 19 (8.3) | 10 (8.5) | 8 (4.6) | 14 (6.5) | 11 (10.5) |
| ≤3 days | 16 (7.0) | 10 (8.5) | 7 (4.0) | 13 (15.3) | 7 (6.7) |
| >3 days and ≤5 days | 3 (1.3) | 0 (0.0) | 1 (0.6) | 1 (1.2) | 4 (3.8) |
| **Solicited systemic AEs** |  |  |  |  |  |
| Fatigue | 33 (14.3) | 20 (17.1) | 33 (19.0) | 11 (12.9) | 23 (21.9) |
| ≤3 days | 30 (13.0) | 17 (14.5) | 28 (16.1) | 10 (11.8) | 19 (18.1) |
| >3 days and ≤5 days | 3 (1.3) | 3 (2.6) | 5 (2.9) | 1 (1.2) | 4 (3.8) |
| Headache | 16 (7.0) | 11 (9.4) | 18 (10.3) | 10 (11.8) | 9 (8.6) |
| ≤3 days | 14 (6.1) | 10 (8.5) | 14 (8.0) | 10 (11.8) | 8 (7.6) |
| >3 days and ≤5 days | 2 (0.9) | 1 (0.9) | 4 (2.3) | 0 (0.0) | 1 (1.0) |
| Myalgia | 17 (7.4) | 3 (2.6) | 17 (9.8) | 8 (9.4) | 9 (8.6) |
| ≤3 days | 16 (7.0) | 3 (2.6) | 15 (8.6) | 8 (9.4) | 6 (5.7) |
| >3 days and ≤5 days | 1 (0.4) | 0 (0.0) | 2 (1.1) | 0 (0.0) | 3 (2.9) |

Supplemental Table 15: Serious AE summary.

|  | **Cohort 1** | | **Cohort 2** | | **Cohort 3** |
| --- | --- | --- | --- | --- | --- |
|  | **V116 (n=230)** | **PCV15 (n=117)** | **V116 (n=174)** | **PPSV23 (n=85)** | **V116 (n=105)** |
|  | n (%) | n (%) | n (%) | n (%) | n (%) |
| **Participants with ≥1 Serious AEs** | 2 (0.9) | 4 (3.4) | 2 (1.1) | 3 (3.5) | 2 (1.9) |
| Cardiac disorders | 1 (0.4) | 2 (1.7) | 0 (0.0) | 0 (0.0) | 0 (0.0) |
| Gastrointestinal disorders | 0 (0.0) | 2 (1.7) | 1 (0.6) | 0 (0.0) | 0 (0.0) |
| Hepatobiliary disorders | 0 (0.0) | 0 (0.0) | 0 (0.0) | 1 (1.2) | 1 (1.0) |
| Infections and Infestations | 1 (0.4) | 1 (0.9) | 0 (0.0) | 0 (0.0) | 1 (1.0) |
| Nervous system disorders | 0 (0.0) | 0 (0.0) | 0 (0.0) | 1 (1.2) | 0 (0.0) |
| Respiratory, thoracic, and mediastinal disorders | 0 (0.0) | 0 (0.0) | 1 (0.6) | 1 (1.2) | 0 (0.0) |
| Vascular disorders | 0 (0.0) | 1 (0.9) | 0 (0.0) | 0 (0.0) | 0 (0.0) |

Supplemental Table 16: List of study investigators.

| **Location** | **Investigator name (Last, first)** | **Location** | **Investigator name (Last, first)** |
| --- | --- | --- | --- |
| Canada | Dionne, Marc | Spain | De luiz Martinez, Gustavo |
|  | Dzongowski, Peter |  | Echave-Sustaeta Maria-Tome, Jose Maria |
|  | Girard, Ginette |  | Masuet Aumatell, Cristina |
|  | Tellier, Guy |  | Narejos Perez, Silvia |
|  | Tytus, Richard |  | Vilella i Morato, Anna |
| France | Jaffuel, Sylvain | Taiwan | Huang, Kuo-Chin |
|  | Nicolas, Jean-Francois |  | Yang, Yi-Ching |
| Israel | Ben Ami, Eytan | United States | Butuk, David J. |
|  | Bendayan, Daniele |  | Cardona, Jose Francisco |
|  | Caraco, Yoseph |  | Daboul, Nizar |
|  | Chowers, Michal |  | Fiel, Thomas |
|  | Darawsha, Mahmud |  | Fraser, Neil J. |
|  | Peer, Avivit |  | Freeman, George Hartley |
| Italy | Blasi, Francesco Bruno |  | Geller, Steven A. |
|  | Castagna, Antonella |  | Harper, Charles Harold |
|  | Costantino, Claudio |  | Johnston, William Henry, Jr. |
|  | Martinelli, Domenico |  | Lenzmeier, Thomas C |
| Japan | Haranaka, Miwa |  | Pelayo, Enrique |
|  | Yono, Makoto |  | Porterfield, Laura |
| South Korea | Choi, Jung Hyun |  | Rigonan, Kathryn R |
|  | Choi, Won Suk |  | Rosen, Jeffrey Bruce |
|  | Lee, Dong-Gun |  | Stacey, Helen L |
|  | Lee, Jacob |  |  |
|  | Shi, Hyejin |  |  |
|  | Song, Joon Young |  |  |
